# Eat Prey, Live: *Dictyostelium discoideum* As a Model for Cell-Autonomous Defenses

**DOI:** 10.3389/fimmu.2017.01906

**Published:** 2018-01-04

**Authors:** Joe Dan Dunn, Cristina Bosmani, Caroline Barisch, Lyudmil Raykov, Louise H. Lefrançois, Elena Cardenal-Muñoz, Ana Teresa López-Jiménez, Thierry Soldati

**Affiliations:** ^1^Faculty of Sciences, Department of Biochemistry, University of Geneva, Geneva, Switzerland

**Keywords:** *Dictyostelium discoideum*, cell-autonomous defense, phagocytosis, autophagy, metal poisoning, nutritional immunity, mononuclear phagocyte system, host–pathogen interactions

## Abstract

The soil-dwelling social amoeba *Dictyostelium discoideum* feeds on bacteria. Each meal is a potential infection because some bacteria have evolved mechanisms to resist predation. To survive such a hostile environment, *D. discoideum* has in turn evolved efficient antimicrobial responses that are intertwined with phagocytosis and autophagy, its nutrient acquisition pathways. The core machinery and antimicrobial functions of these pathways are conserved in the mononuclear phagocytes of mammals, which mediate the initial, innate-immune response to infection. In this review, we discuss the advantages and relevance of *D. discoideum* as a model phagocyte to study cell-autonomous defenses. We cover the antimicrobial functions of phagocytosis and autophagy and describe the processes that create a microbicidal phagosome: acidification and delivery of lytic enzymes, generation of reactive oxygen species, and the regulation of Zn^2+^, Cu^2+^, and Fe^2+^ availability. High concentrations of metals poison microbes while metal sequestration inhibits their metabolic activity. We also describe microbial interference with these defenses and highlight observations made first in *D. discoideum*. Finally, we discuss galectins, TNF receptor-associated factors, tripartite motif-containing proteins, and signal transducers and activators of transcription, microbial restriction factors initially characterized in mammalian phagocytes that have either homologs or functional analogs in *D. discoideum*.

## Introduction

The mononuclear phagocyte system (MPS), comprising monocytes, macrophages, and dendritic cells, is the first line of defense against infection. MPS cells monitor blood and tissue for the presence of microbes and respond to conserved molecules produced by and/or to damage caused by potential pathogens. These signals trigger innate-immune pathways that activate cell-autonomous defense mechanisms and secretion of intercellular signals that orchestrate inflammatory and subsequent adaptive immune responses.

Cell-autonomous defense mechanisms, which exist in MPS cells and also non-immune cells, provide a rapid antimicrobial response and evolved from the predator–prey relationship among the ancestors of current eukaryotes and prokaryotes (1, 2). In MPS cells, cell-autonomous defenses include phagocytosis and autophagy, processes that originated in ancient eukaryotes for nutrient acquisition and reallocation. Indeed, the basic components of these pathways are conserved in immune cells of all metazoa and in bacterivorous and fungivorous single-celled eukaryotes such as amoebae. Microbial mechanisms to subvert cell-autonomous defenses predate metazoa and likely originated as resistance to predation by single-celled eukaryotes. Given this evolutionary context, amoebae are relevant surrogate phagocytes for the study of the conserved innate-immune responses of MPS cells and of microbial mechanisms to resist killing. Although the infection of amoebae cannot completely recapitulate the complexities of host–pathogen interactions in metazoa, the innate defenses of MPS cells establish the critical initial immune response to microbes, and MPS cells are often the physical interface of host and intracellular pathogens. Valuable insight can thus be gained by studying the interactions between microbes and amoebae that rely solely on cell-autonomous defenses for survival.

In this review, we focus on the use of the soil dwelling, social amoeba *Dictyostelium discoideum* as a model phagocyte to study the interactions between intracellular pathogens and cell-autonomous defense mechanisms of MPS cells. We cover phagocytosis with an emphasis on phagosome maturation and the mechanisms used to create an antimicrobial environment within the phagosome. These mechanisms include the delivery of lytic enzymes, the generation of reactive oxygen species (ROS), and the manipulation of the concentrations of divalent metals to either poison microbes or inhibit their metabolic activity. We describe autophagy as a defense pathway that responds when phagocytosis is insufficient to eliminate infection. We also highlight microbial interference with these defense responses first elucidated using *D. discoideum*. Finally, we discuss microbial restriction factors, initially characterized in MPS cells, that have either sequence homologs or potentially functional analogs in *D. discoideum*. Determining their contribution to cell-autonomous defense mechanisms in this amoeba will further strengthen its usefulness as a model for host–pathogen interactions.

## Predation Resistance, Virulence, and Cell-Autonomous Defenses

Given the complex interactions between pathogenic microorganisms and their metazoan hosts, it is difficult to pinpoint the origins of microbial virulence. How would a naïve bacterium encountering an elaborate immune response for the first time persist long enough to evolve mechanisms to counteract antimicrobial defenses? On the host side, why are such extensive immune responses in place? When did it all begin? An emerging concept in host–pathogen interactions is that microbial virulence evolved from selective forces in the environment such as the pressure to resist predation by amoebae and other protozoa (3–6).

In addition to competing for nutrients and adapting to variations in environmental conditions such as temperature and moisture, successful microbes avoid being a meal for predatory protozoa. These resistance mechanisms include avoidance of phagocytosis, e.g., masking of the microbial surface and biofilm formation, avoidance of digestion, e.g., inhibition of phagosome maturation, escape from the phagosome, the killing of the predator by toxin secretion pre- or post-phagocytosis, and the use of specialized secretion systems (3). On the predator side, the advent of resistance in turn selected for more robust strategies to kill bacteria and/or counteract this resistance. This ancient fight for survival among single-celled species with short generation times and large population sizes provided the context for the evolution of virulence and cell-autonomous defenses. As metazoa evolved and gained complexity, these defenses were conserved and also expanded (7, 8). Concurrently, some microbes evolved mechanisms not just to avoid killing but to survive inside hosts and exploit them for resources.

The coevolution of host and microbes beginning at the single-celled stage provides a plausible explanation for the conservation of cell-autonomous defenses. It is important to remember that single-celled eukaryotic predators continued to evolve and that successful cell-autonomous defense strategies are present in extant species such as *D. discoideum*. Bacteria must therefore contend with similar defense mechanisms in macrophages and amoebae; consequently, predation selects for bacterial survival strategies that are likely to resist killing by MPS cells (9–12). Another consequence of this conservation is that amoebae can select for and act as reservoirs of microbes that can infect humans, including *Mycobacterium* species, *Legionella pneumophila*, and *Vibrio cholera* (13–15). This evolutionary history makes amoebae ideal, relevant model phagocytes to study host–pathogen interactions (16–19).

## *Dictyostelium* *discoideum*

**Figure 1:**
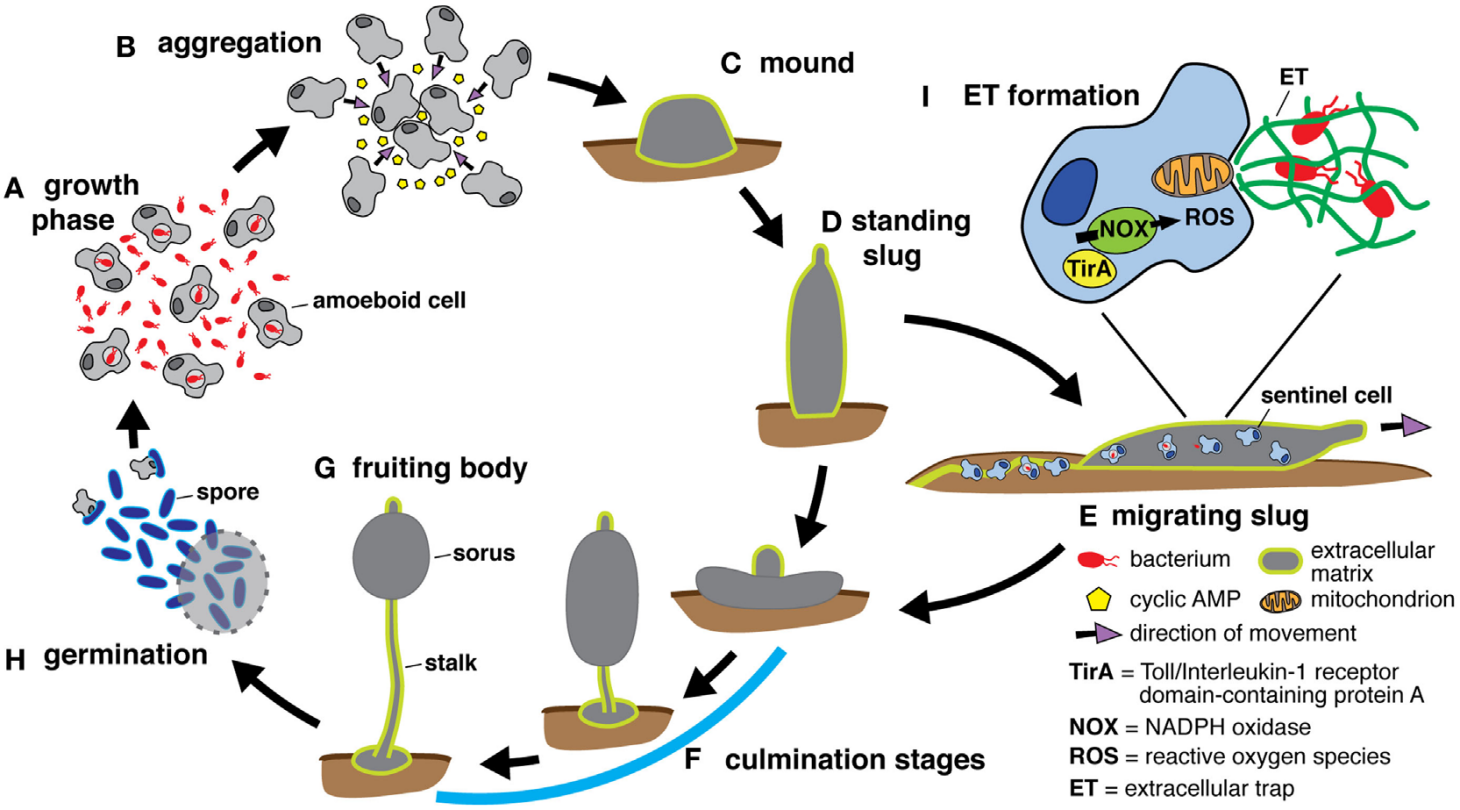
The *Dictyostelium discoideum* life cycle includes multicellular stages. **(A)** During the growth phase of development, amoeboid cells feed on bacteria and replicate by binary fission. The development cycle is initiated upon resource depletion, and aggregation occurs when starving cells secrete cyclic AMP to recruit additional cells **(B)**. The aggregating cells organize to form the mound stage enclosed within an extracellular matrix composed of cellulose and mucopolysaccharide (26) **(C)** and continue to develop into the standing slug **(D)**. Depending on its environment, the standing slug either falls over to become a migrating slug that moves toward heat and light **(E)** or proceeds directly to the culmination stages **(F)** that ultimately produce the fruiting body, which consists of a spore-containing structure, the sorus, held aloft by a stalk of dead cells **(G)**. Spores are released from the sorus and germinate into growing cells **(H)**. Under optimal conditions, the developmental cycle takes around 24 h. If the slug forms underground, it migrates toward the surface to maximize spore dissemination. To protect itself from infection during migration, the slug possesses a rudimentary immune system comprising phagocytic sentinel cells. These cells move throughout the slug, take up bacteria and toxins, and are shed along with extracellular matrix as the slug moves **(E)**. In response to bacteria, sentinel cells release extracellular traps, derived from mitochondrial DNA, *via* an unknown mechanism involving NADPH oxidase (NOX)-generated reactive oxygen species (ROS) and TirA, a soluble protein containing a toll/interleukin 1 receptor domain **(I)**.

*Dictyostelium discoideum* belongs to the Amoebozoa phylum, which diverged from the Opisthokonts, the phylum to which the animals belong, after sharing a common ancestor with plants. During its growth phase, the amoeba replicates by binary fission and employs phagocytosis to kill and extract nutrients from bacteria in the soil. Upon nutrient depletion, starvation induces amoebae to undergo a developmental cycle in which approximately 100,000 cells aggregate by chemotaxing toward cyclic AMP, differentiate into multiple cell types, and transition through several multicellular stages to ultimately produce the fruiting body that comprises a spore-containing sorus resting upon a stalk of dead cells (Figure 1) (20–22). Depending on environmental conditions, the early multicellular stage continues to develop in place or transitions to a motile slug that migrates away from ammonia and toward heat, light, and oxygen before fruiting body culmination (20–25). When development is initiated underground these cues guide the slug to the soil surface, where spore dispersion is more likely.

Due to its multicellular cycle, *D. discoideum*, referred to as a social amoeba, is used as a model to study aspects of development including intercellular signaling (27) [reviewed in Ref. (22)], quorum sensing (28), cell–cell recognition (29), cell fate determination (30), tissue patterning (31), and even societal concepts such as cooperativity and altruism (32). It is also an established model for basic cell biological processes, including phagocytosis (33), macropinocytosis (34), chemotaxis (35), autophagy (36), and oxygen sensing (37), and the functions of proteins implicated in human diseases including Alzheimer’s and Parkinson’s (38–41). Indeed, this amoeba has proven to be a versatile model organism and is gaining traction as an attractive alternative to animal models (42, 43).

*Dictyostelium discoideum* has been used as a host cell for *L. pneumophila* (44–47), *Mycobacterium* species (48–51), *V. cholera* (17), *Francisella noatunensis* (52, 53), *Pseudomonas aeruginosa* (16), *Salmonella enterica* (54), and other intracellular pathogens (55). Moreover, its genome encodes numerous homologs of proteins and protein domains involved in sensing and responding to microbes by macrophages (5) (http://dictybase.org). The conservation of macropinocytosis, chemotaxis, phagocytosis, and autophagy pathways in *D. discoideum* make it a model MPS cell. Here we focus on phagocytosis and autophagy within the context of cell-autonomous defenses. Macropinocytosis and chemotaxis are beyond the scope of this review and have been covered extensively elsewhere [for reviews on chemotaxis (56, 57); for reviews on macropinocytosis (58, 59)].

From a practical standpoint, its amenability to experimentation also makes *D. discoideum* an ideal model organism. It is easily cultivatable and can be grown axenically in liquid media, which enables analysis of mutant strains defective for growth on bacteria. Cultures can be readily scaled up for biochemical and cell biological techniques (43) as well as high-throughput genetic and drug discovery screens (60–62). It is also well suited for microscopy including live-cell imaging (63). An extensive molecular genetic toolkit has been developed for the generation of mutants and ectopic gene expression (64). The haploid genome of multiple strains and closely related species have been sequenced (65–68), and numerous RNAseq and transcriptomic analyses have been performed (69, 70). The community’s online resource, dictyBase, provides a central location to access sequence data, techniques, and available mutants and plasmids (71) (http://dictybase.org). Relevant its use as a model phagocyte, there are established protocols for infecting *D. discoideum* with various bacterial pathogens (63, 72, 73) and for monitoring and quantifying autophagy (74).

## Phagocytosis

**Figure 2:**
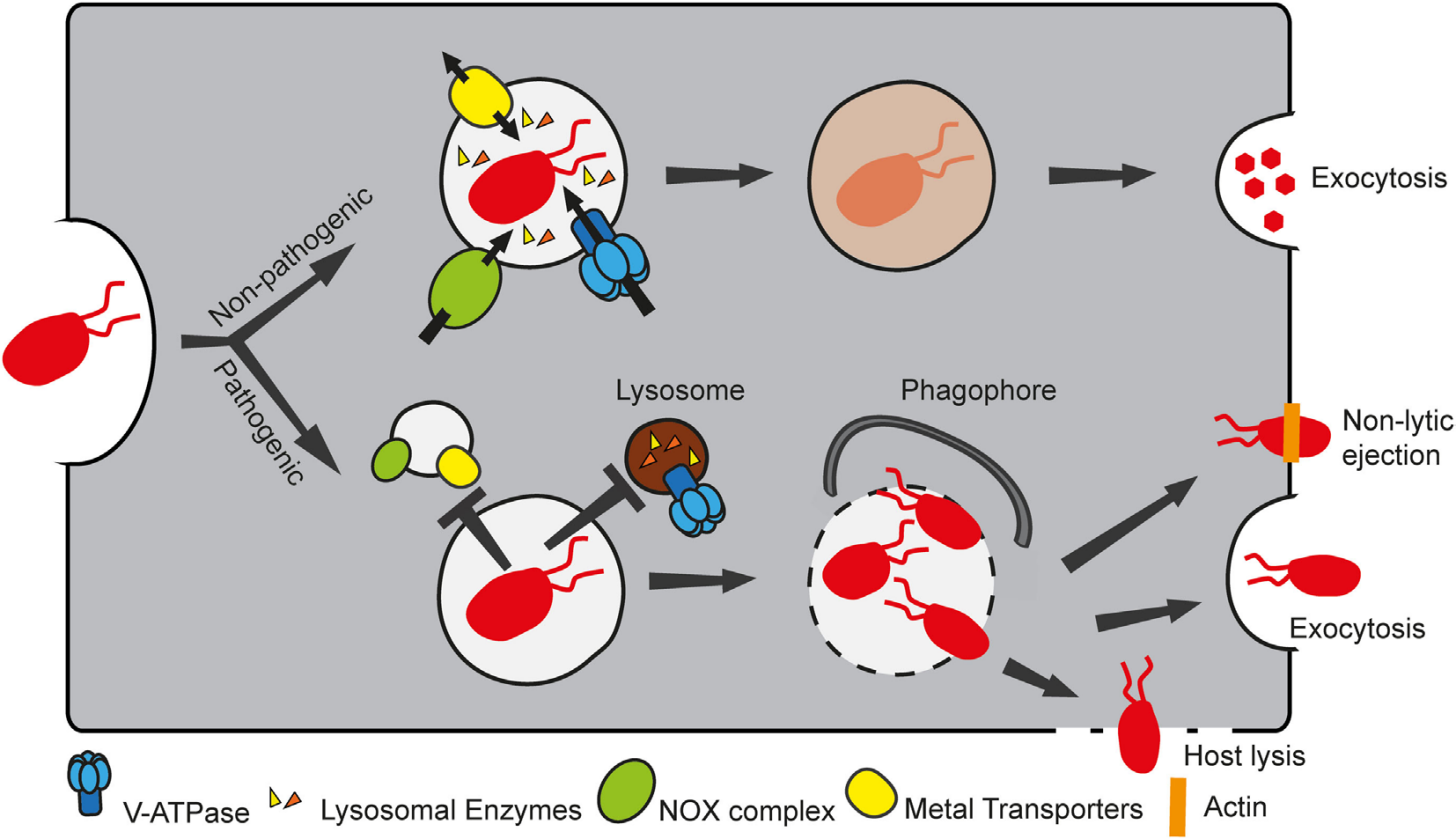
Non-pathogenic and pathogenic bacteria follow different fates in *Dictyostelium discoideum*. *D. discoideum* takes up bacteria by phagocytosis. Non-pathogenic food bacteria follow the normal phagosomal maturation pathway, whereby the phagosome acquires several components, including the vacuolar ATPase (V-ATPase), lysosomal enzymes, the NADPH oxidase (NOX) complex, and several metal transporters to create a microbicidal compartment that digests and kills bacteria. Intracellular pathogens, however, are able to manipulate the maturation program, by preventing the phagosome from becoming bactericidal, thus ensuring proliferation in a “friendly” compartment. In addition, certain pathogens can eventually escape the compartment. In this case, they can either be recaptured by autophagy, or exit the host cell by exocytosis, or by lytic or non-lytic processes.

Phagocytosis is the process that allows engulfment of particles larger than 200 nm and is used by MPS cells to ingest and kill pathogens as well as to activate the adaptive immune response through antigen presentation. The phagocytosis maturation pathway is highly conserved between MPS cells and *D. discoideum*, which uses the process for feeding (8). In a simplified view, the particle to ingest is recognized by surface receptors, and this interaction triggers a signaling cascade that stimulates polymerization of actin to deform the membrane around the particle. After closure of the phagocytic cup, the newly formed phagosome undergoes maturation, a series of steps necessary to render the phagosome a highly acidic, degradative and oxidative compartment (Figure 2). Many pathogens, such as *L. pneumophila, S. enterica*, and *Mycobacterium* spp., have evolved ways to escape the phagosome or subvert its maturation to replicate within the host cell. In this section, we will briefly summarize the phagocytosis maturation steps in *D. discoideum* and how this model phagocyte has been used to extend our knowledge of several infectious diseases. It should be noted that studies using *D. discoideum* as a model phagocyte have mainly been performed with axenic laboratory strains. These strains are able to grow in the absence of bacteria due to a null mutation in the gene encoding the Ras-regulating neurofibromin, which results in enlarged macropinosomes that facilitate uptake of sufficient nutrients from liquid media to support growth and also enables the mutants to phagocytose larger particles than wild-type strains (75). Effects of this mutation on phagosome maturation have not been reported.

### Particle Recognition and Phagocytosis Initiation

Innate-immune cells can recognize several pathogen-associated molecular patterns (PAMPs) secreted or present at the bacterial cell wall *via* specific pathogen recognition receptors (PRRs). In mammals, these include toll-like receptors (TLRs), integrins, scavenger receptors, and C-type lectins (76). TLRs monitor the cell surface and endocytic compartments and activate cell-autonomous defenses upon detection of PAMPs, while lectins, scavenger receptors, and integrins function as phagocytic receptors that bind to particles and are able to trigger uptake even in non-phagocytic cells (76). TLRs contain ligand-binding leucine-rich repeats (LRRs) in the luminal/extracellular domains and a toll/interleukin 1 receptor (TIR) domain in the cytoplasmic tail that mediates protein–protein interactions. *D. discoideum* does not have TLRs, but two cytosolic proteins with TIR domains, TirA and TirB, have been identified. Depletion of TirA inhibits growth on a laboratory strain of *Klebsiella* used as a food bacterium, but not in media (77). How TirA is involved in sensing and/or induction of phagocytic uptake remains to be studied. The *D. discoideum* genome encodes >150 LRR-containing proteins, but whether they function as PRRs remains to be determined (5).

In *D. discoideum*, only a few phagocytic receptors have been molecularly identified so far (5). The most studied are the integrin-like Sib (similar to integrin-β) family of proteins, comprising five members (SibA-E). Like human integrins, they contain a von Willebrand factor type A and a glycine-rich transmembrane domain and can interact with the actin-binding protein talin (78). Two members of the family, SibA and SibC, are directly involved in adhesion to substrate and to particles (78). Phg1a, a member of the TM9 family, and SadA were previously identified as phagocytic receptors (79, 80); however, recent findings show that these two proteins are instead involved in regulating surface levels of SibA (81).

Lectins and scavenger receptors might also function as phagocytic receptors in *D. discoideum*. Three homologs of scavenger receptor class B proteins in mammals, LmpA, LmpB, and LmpC, are present in *D. discoideum*. LmpB is found on lipid rafts at the plasma membrane and in early phagocytic compartments and may function as a phagocytic receptor (82–84). It is thought that *D. discoideum* also possesses lectin-like receptors, as it was shown to be able to bind specifically to certain sugar derivatives (85, 86).

A well-studied chemoattractant and phagocytosis stimulator for *D. discoideum* is folate, a metabolite secreted by certain bacteria. Recently, Pan and colleagues identified fAR1, a G-protein coupled receptor for folic acid involved in signaling and initiation of uptake but not binding of bacteria (87). Presumably, the cytoskeletal rearrangements downstream of fAR1 that facilitate chemotaxis can also initiate phagocytosis when a burst of fAR1 activation occurs around a bacterium that is a concentrated source of folate. *D. discoideum* sensing of bacterial capsule independent of folate has also been described but not further characterized (88).

### Actin Dynamics and Phosphatidylinositol Phosphates (PIPs)

After binding of ligands to their receptors, heterotrimeric G proteins are involved in downstream signaling to initiate phagocytosis through F-actin rearrangements. In *D. discoideum*, the G4αGβγ complex has been proposed to be activated by the fAR1 folate receptor, was shown to be implicated in particle uptake, and is therefore the most likely candidate involved in signaling from the phagocytic receptors to drive actin polymerization (87, 89). F-actin rearrangements occur at the uptake site to drive formation of pseudopods and the phagocytic cup. F-actin polymerization at the uptake site is driven by the Arp2/3 complex, an actin nucleation factor, and its activator, the SCAR/WAVE complex, in both macrophages and *D. discoideum* (90, 91). In addition, several other actin-binding proteins, such as profilins, cofilins, and Abp1, are present during the formation of the phagocytic cup [(92, 93), for a more detailed review on the phagocytic process in *D. discoideum*, see Ref. (94)]. Regulation of actin dynamics and subsequent trafficking events by Rho GTPases is also conserved in *D. discoideum*, with Rac1 homologs (RacA, B, C, and G) thought to be the main regulators of phagocytic uptake. Notably, Rac1 is involved in FcγR-mediated phagocytosis in macrophages [for a comprehensive review on Rho signaling, see Ref. (95)].

Phosphatidylinositol phosphates are important players during phagocytic uptake and maturation because they provide an identity to each compartment. PIP dynamics are well conserved between macrophages and *D. discoideum*. Briefly, phosphatidylinositol (4,5)-bisphosphate PI(4,5)P_2_ is the predominant PIP of the plasma membrane and is involved in recruiting and activating actin-binding proteins and nucleation-promoting factors. After receptor engagement, PI(4,5)P_2_ is phosphorylated into PI(3,4,5,)P_3_ by phosphatidylinositol 3-kinase and hydrolyzed into diacylglycerol and inositol (1,4,5)-trisphosphate, second messengers involved in calcium release and activation of further signaling cascades, by the phospholipase C kinase. Decrease of PI(4,5)P_2_ around the uptake site is necessary to then allow actin disassembly and closure of the phagocytic cup. In addition, closure of the phagocytic cup requires Dd5P4, the *D. discoideum* homolog of the phosphatase OCRL, which dephosphorylates PI(3,4,5)P_3_ into PI(3,4)P_2_ (96–98). Extensive recycling of plasma membrane components, including adhesion molecules, is a common feature shared by *D. discoideum* and macrophages in the early phases of phagosome formation, and in both organisms this step is regulated by the WASH complex, an Arp2/3 activator (99–101).

### Phagosome Maturation

**Figure 3:**
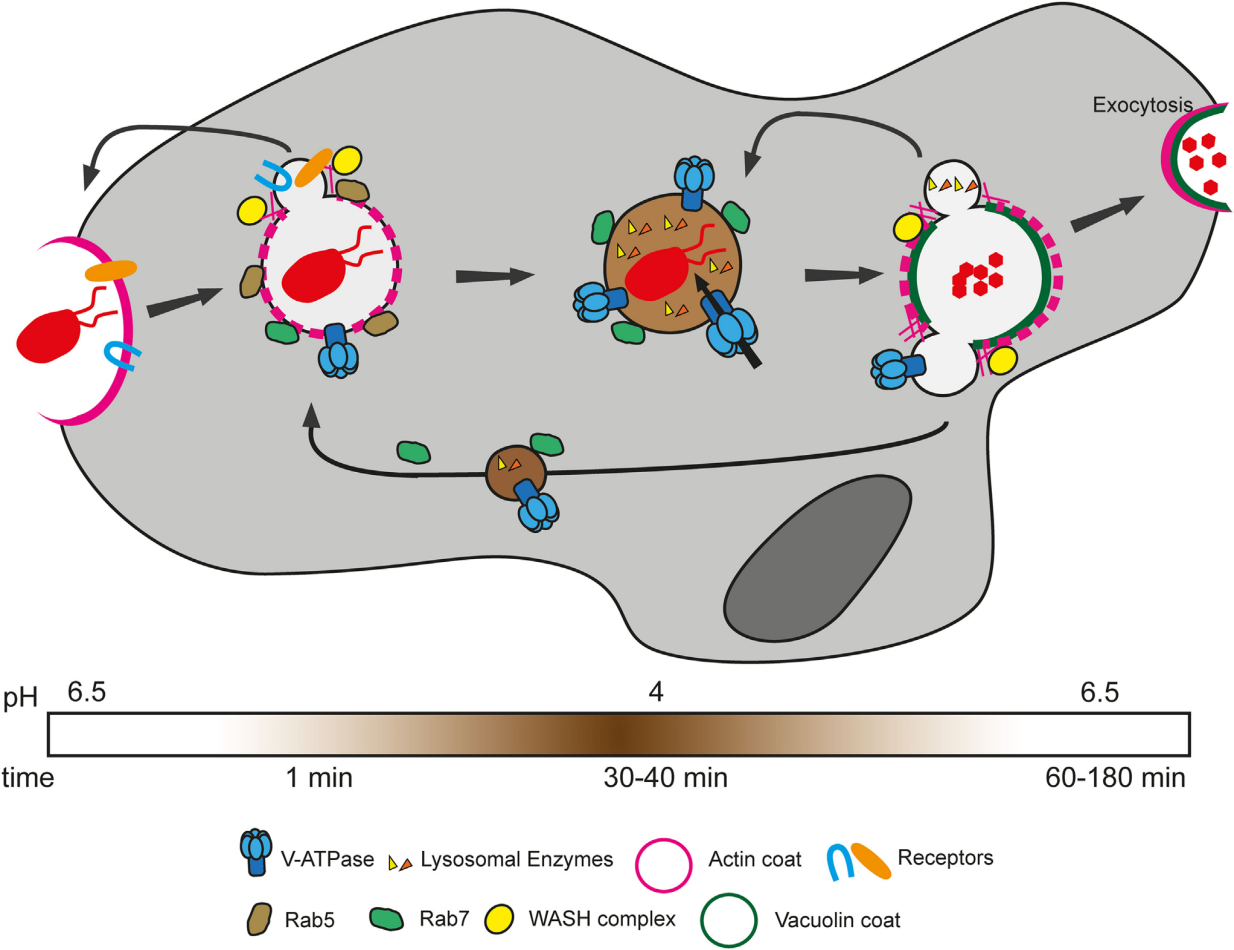
Phagosome maturation in *Dictyostelium discoideum*. Bacteria are recognized and sensed by various phagocytic and/or signaling receptors. This triggers signaling cascades that allow actin polymerization and deformation of the membrane to engulf the particle. After closure of the phagosome, bacteria are enclosed in an early phagosome, which gradually loses its actin coat and is characterized by the presence of Rab5. As early as 1 min after uptake, Rab7 is recruited to the phagosome, enabling fusion with lysosomes. Meanwhile, phagocytic receptors and plasma membrane proteins are recycled to the cell surface through actin polymerization induced by the WASH complex through Arp2/3 activation. The proton pump vacuolar ATPase (V-ATPase) is also acquired early in the maturation, ensuring rapid decrease of the luminal pH. Lysosomal enzymes, comprising proteases, are acquired in subsequent waves of delivery and function at low pH to degrade bacterial components. After about 40 min, the V-ATPase and lysosomal enzymes are recycled by the WASH complex in separate waves of recycling. Finally, non-digested bacterial remnants are expelled by exocytosis.

After closure of the phagocytic cup, the ingested particle is found in a closed compartment termed the phagosome. Extensive proteomic analyses as well as more recent live microscopy experiments have demonstrated the extraordinary plasticity of this organelle and the high degree of conservation of the phagosome maturation pathway between mammals and *D. discoideum* (8, 84). Phagosome maturation is a well-orchestrated series of events, which ensures killing and digestion of ingested bacteria (Figure 3). In a simplified view, Rab GTPases, notably Rab5 and Rab7, act as the masterminds of phagosome maturation by sequentially recruiting effectors involved in the various maturation steps [for an extensive review, see Ref. (102)]. In macrophages, Rab5 and its effectors are responsible for docking and fusion of endocytic compartments with the nascent phagosome and for acquisition of early phagosomal markers (103, 104). Subsequently, Rab7 ensures fusion with late-endosomal/lysosomal compartments and thus delivery of the lysosomal digestive content into the phagosome (104, 105). Rab GTPases are highly conserved at the protein sequence level between mammals and *D. discoideum*; indeed, this amoeba has homologs of most mammalian Rab GTPases that have been reported to be associated with phagosomes (102). *D. discoideum* Rab7 is recruited as early as 1–3 min after phagosome closure and regulates delivery of lysosomal proteins (106, 107). The localization and function of *D. discoideum* Rab5 have not been reported.

Recruited within minutes to the phagosome, the H^+^-vacuolar ATPase (V-ATPase) is the main agent of acidification and pumps protons inside the phagosome thanks to ATP hydrolysis (108). By creating a proton gradient, the V-ATPase is a crucial complex not only for the killing and digestion of bacteria but also for the progression of the maturation program. Notably, the proton gradient is necessary for proper delivery and activity of lysosomal enzymes, as well as for the function of ion transporters, involved in poisoning by or deprivation of metals (detailed below). The V-ATPase has been shown to be delivered by fusion with lysosomal vesicles or tubules in *D. discoideum* and macrophages, respectively (108–110). Rapid acidification of the compartment ensues, with the lowest pH reached between 10 and 30 min after phagosome formation, depending on the measuring method (93, 111). In contrast to macrophages, whose phagosomes were reported to reach a pH of 4.5–5, *D. discoideum* phagosomes are more acidic, with a pH as low as 3.5–4 (111–113).

Although unprocessed antigens have been shown to be regurgitated from late-endosomal compartments in dendritic cells to allow antigen uptake by other MPS cells (114, 115), in general, the acidic phagosome of MPS cells is thought of as a dead-end for ingested bacteria. In contrast, the acidic *D. discoideum* phagosome matures into a postlysosome. The V-ATPase and lysosomal enzymes were shown to be retrieved in several subsequent waves of recycling mediated by the WASH complex, which drives local actin polymerization (116, 117). The WASH complex is a nucleation-promoting factor necessary for the activation of the Arp2/3 complex and actin polymerization in both mammals and *D. discoideum* (99, 100, 117). During retrieval of the V-ATPase, the phagosome reaches its neutral pH, and progressively acquires the postlysosomal markers vacuolin A and B, homologs of the mammalian lipid raft-associated flotillins (118–120). The postlysosome is also characterized by the presence of an actin coat and the actin-binding protein coronin (118). This compartment then fuses with the plasma membrane in a mechanism akin to exocytosis to expel its non-digested materials. Interestingly, this process is reminiscent of exocytosis of secretory lysosomes in mammalian cytotoxic cells of the immune system (121). In mammals, lysosomes were shown to fuse with the plasma membrane upon increase of cytosolic Ca^2+^ concentration. In *D. discoideum*, Ca^2+^ was also shown to be involved in exocytosis, with mucolipin, a Ca^2+^ transporter, involved in regulating this process. Mucolipin is thought to pump Ca^2+^ inside postlysosomes, thereby inhibiting fusion with the plasma membrane by decreasing local Ca^2+^ concentration (122).

### Microbial Manipulation of *D. discoideum* Phagocytosis

Certain intracellular bacterial pathogens are known for subverting phagosome maturation to prevent the formation of an unfriendly bactericidal compartment and to enable replication within the host cell. *L. pneumophila*, a Gram-negative bacterium that causes Legionnaire’s disease, is able to arrest early phagosomal maturation. In fact, the V-ATPase and other early endocytic markers are not delivered to the *Legionella*-containing vacuole (LCV) in either *D. discoideum* or macrophages (123). The endoplasmic reticulum (ER) is recruited in proximity to and fuses with the LCV, which becomes enriched in ER markers such as calnexin and calreticulin. This enrichment of ER markers is a consequence of a major strategy used by *L. pneumophila* to proliferate inside the host cell, which is termed identity theft and consists of changing the identity of its compartment to resemble the ER by recruiting different GTPases and PIPs (124, 125). For example, thanks to several bacterial effectors secreted through its type 4 secretion system, this pathogen is able to change the PIP composition of the phagosome by notably acquiring PI(4)P, a PIP normally associated with the *trans*-Golgi, ER, and plasma membrane (47). Other proteins involved in PIP dynamics and phagosome maturation, such as phosphatidylinositol 3-kinase and Dd5P4 have been shown to restrict *L. pneumophila* growth in *D. discoideum* (45, 46). *D. discoideum* has been used successfully to isolate and characterize the proteome of LCVs (44, 126). These studies have highlighted the importance of ER/Golgi small GTPases, such as Arf1 and Rab1, in gradually modifying the identity of the LCV. Rab8, a *trans*-Golgi-associated Rab GTPase, has also been detected at the LCV membrane and been shown to play a role in the association of SidC, a bacterial effector that mediates ER recruitment (44). Interestingly, Hoffmann and colleagues compared the LCV proteomes purified from murine macrophages and *D. discoideum* and uncovered that, if only considering proteins with conserved roles in these two organisms, about 50% of LCV-associated proteins were found in both organisms (126). These include the aforementioned Arf1, Rab8, and Rab1, as well as proteins involved in lipid metabolism, suggesting that *L. pneumophila* uses similar mechanisms to replicate in *D. discoideum* and macrophages and further corroborating the case that *D. discoideum* is an excellent model to study *L. pneumophila* infection.

Like *Legionella pneumophila*, albeit with completely different strategies, *Mycobacterium* spp. manipulate the phagosome maturation pathway. In fact, *Mycobacterium tuberculosis*, the causative agent of tuberculosis, and *Mycobacterium marinum*, a closely related mycobacterium that infects frogs and fish, prevent acquisition of the V-ATPase in macrophages (127). This was confirmed in *D. discoideum*, where it was shown that *M. marinum* is able to prevent accumulation of the V-ATPase and of cathepsin D (48). Recently, it was proposed that the WASH complex plays a role in preventing association of the V-ATPase with the mycobacteria-containing vacuole (MCV) by inducing polymerization of actin around the MCV, which probably prevents fusion with acidic vesicles. This mechanism was first studied in *D. discoideum*, but further confirmed in human macrophages with both *M. tuberculosis* and *M. marinum* (128). Of note, the mechanism of escape of cytosolic *M. marinum* from the host cell, termed ejection, and of cell-to-cell spreading has been well characterized and studied using *D. discoideum* as a host model [(49); reviewed in Ref. (129, 130)]. Interestingly, *D. discoideum* has also been extensively used as a model phagocyte to screen for new mycobacterial virulence factors (131–134).

## Microbicidal Phagosome

*Dictyostelium discoideum* and macrophages employ conserved strategies to kill bacteria. As discussed previously, the V-ATPase has a central role in phagosome acidification; however, a low pH is not sufficient *per se* to kill bacteria. In fact, the phagosome acquires a series of proteases, hydrolases, lysozymes, and antimicrobial peptides necessary to breakdown several bacterial components or disrupt membrane integrity. Moreover, microbes can be poisoned and killed by transport of certain metals or by the production of ROS inside the compartment. Furthermore, metals can be pumped out of the phagosome to prevent bacterial growth. These bacterial-control strategies will be described in this section.

### Lysozymes and Lysosomal Enzymes

Lysozymes, glycosidases that digest the peptidoglycan layer present in the cell wall of bacteria, belong to the Aly family in *D. discoideum* (135). Upregulation of lysozyme expression differs depending on the bacteria used as food. *D. discoideum* grown on Gram-positive bacteria upregulate AlyA, AlyB, AlyC, and AlyD whereas growth on Gram-negative bacteria leads to an increase in AlyL expression (136).

Lysosomal enzymes comprise several classes of enzymes involved in hydrolysis of sugar groups, such as mannosidases, or peptide bonds, such as proteases, and have been involved extensively in resistance to certain pathogens as well as bacterial killing in MPS cells. For instance, cysteine and serine cathepsins have been implicated in resistance to and killing of *Staphylococcus aureus* in neutrophils and macrophages (137, 138). More recently, *M. tuberculosis* was shown to downregulate expression and inhibit activity of cysteine proteases in macrophages, thus ensuring its replication (139). Cathepsin D, an aspartic protease, was also involved in resistance to *Listeria monocytogenes*, an intracellular food-borne pathogen (140).

Two main classes of lysosomal enzymes, bearing different posttranslational modifications, have been characterized in *D. discoideum*. The first class includes α-mannosidase, β-glucosidase, and cathepsins and is modified with mannose-6-phosphomethyldiester and/or mannose-6-sulfate, also known as common antigen-1 (141, 142). The second class of enzymes contains an *N*-acetylglucosamine-1-phosphate and comprises cysteine proteases (143). These different classes of enzymes reside in different vesicles at the steady state level and are recruited to phagosomes in a sequential manner, with first a wave of cysteine proteases followed by enzymes bearing the mannose-6-sulfate modification (84, 144).

Although lysosomal enzymes appear to be involved in bacterial killing in *D. discoideum* as in macrophages, direct evidence is lacking. Deletion of the cathepsin D gene is not sufficient to abolish growth on the food bacterium *Klebsiella* (142). Several *D. discoideum* mutants impaired in lysosomal enzyme trafficking and/or activity have been characterized including strains lacking WshA, a subunit of the WASH complex involved in lysosomal enzyme recycling (117), LvsB, a protein involved in restricting heterotypic fusion of early endosomes with postlysosomal compartments (145), and TM9 protein A, which is involved in the sorting of glycosidases, cathepsins, and lysozymes (146). Interestingly, these mutants exhibit growth defects specific to certain subsets of bacterial species (117, 145–148). These data suggest that different classes of lysosomal enzymes might play redundant roles, that they are not the sole killing strategy employed by *D. discoideum*, and that specific mechanisms may be used depending on the encountered bacteria.

### Reactive Oxygen Species

Reactive oxygen species are key components of cell-autonomous defenses of MPS cells and function as antimicrobial effectors (149) as well as signaling molecules that regulate NF-κB (150, 151), autophagy (152), cytokine secretion (153), inflammasome activation (154), and apoptosis (155). ROS are implicated in the regulation of pH within phagosomes and the production of antigenic peptides in dendritic cells (156–158). ROS have also been implicated in the regulation of cytoskeleton dynamics and chemotaxis (159, 160). The major source of ROS in MPS cells is the NADPH oxidase (NOX) 2. Depending on its localization, NOX2 generates superoxide by transferring an electron from cytosolic NADPH to O_2_ in either the extracellular space or the lumen of the phagosome. Subsequent reactions convert superoxide into additional ROS. Superoxide dismutase catalyzes its conversion to hydrogen peroxide, which in turn reacts with Fe^2+^ in the Fenton reaction to generate hydroxyl radicals or with Cl^−^ to produce hypochlorous acid *via* myeloperoxidase (161–163).

NOX2 is a heterodimer comprising the transmembrane proteins gp91^phox^/Nox2, the catalytic subunit, and p22^phox^, the regulatory subunit. NOX2 activation occurs downstream of extracellular receptors including integrins and Fc receptors and is coupled with phagocytosis. Activation requires three additional subunits, p67^phox^/neutrophil cytosol factor (Ncf) 2, p40^phox^/Ncf4, and p47^phox^/Ncf1, which form a ternary complex in the cytosol that is recruited to membrane-localized NOX2 by the small GTPases Rac1 and 2 (161–163). Mutations in NOX2 subunits cause chronic granulomatous disease (CGD), a condition that makes patients susceptible to recurring bacterial and fungal infections and demonstrates the importance of the NOX2-generated oxidative burst in the immune response (164, 165). Mitochondrial ROS production activated downstream of TLR signaling also contributes to antimicrobial mechanisms (166, 167).

Because ROS can damage host and microbe alike, ROS production and localization are tightly regulated, and MPS cells express ROS detoxifying enzymes such as superoxide dismutases (SODs), catalases, and peroxiredoxins to prevent self-damage. Microbes that persist inside the phagosome have mechanisms to minimize oxidative stress. These include expression of robust systems to maintain internal redox homeostasis (168), secretion of SODs and catalases to detoxify their compartment (149, 169), and deployment of effectors that inhibit NOX2 activation and/or delivery to the phagosome (170–173).

The *D. discoideum* genome encodes three catalytic NOX subunits: NoxA and NoxB, which are homologs of gp91^phox^/Nox2, and NoxC, which is a homolog of Nox5 (174–176). It also encodes one homolog of p22^phox^, CybA, and NcfA, a homolog of the cytosolic activating factor p67^phox^/Ncf2 (174, 175). RNAseq data indicate that NoxA, CybA, and NcfA are expressed during growth while NoxB and NoxC are mainly expressed during development (69, 174). However, long-term growth on *Klebsiella* can cause upregulation of NoxB in growing cells (136). Interestingly, *D. discoideum* expresses multiple SOD and catalase homologs (177–179) and exhibits a high resistance to oxidative stress (180), which suggests that it encounters internally and/or externally generated ROS regularly.

Whether ROS contribute to cell-autonomous defenses during the growth phase of *D. discoideum* is not clear. Mutants lacking *noxA* or both *noxA* and *noxB* exhibit no growth or killing defects when grown on *Klebsiella* (148, 174). This lack of a defect might be a consequence of redundant killing mechanisms or of challenge with a bacterium that is easily killed. Intriguingly, a *D. discoideum* mutant lacking the Xpf nuclease, a component of DNA damage repair machinery, accumulates more mutations when grown on a range of bacteria including *Klebsiella* than when grown axenically in media (181). One possible explanation is that Xpf is required to repair DNA damaged by ROS generated in response to bacteria.

Excessive ROS production in growing cells due to a deletion of a surface-localized SOD causes defects in chemotaxis and cell motility *via* sustained Ras activation (182, 183). The authors hypothesize that the chemotaxis defect prevents the inclusion of the cell in the multicellular stage and thus prevents propagation of the potentially mutagenized genome. It is tempting to speculate that excessive ROS production in response to a resistant microbe causes the same effect and inhibits the inclusion of an infected cell in the multicellular stages. Although the context is different, this “self-sacrifice” might be analogous to ROS-induced apoptosis of infected macrophages (155, 184).

Reactive oxygen species have functions during development. Extracellular ROS scavengers inhibit aggregation (185), and mutations in the individual NOX subunit genes, *noxA, noxB, noxC*, or *cybA*, or in the development stage-specific catalase gene, *catB*, cause defects in fruiting body formation when developed under axenic conditions (174, 186). These results indicate a signaling role for ROS. When developed after feeding on bacteria, a *no*x*ABC^−^* triple mutant exhibits increased bacterial contamination of fruiting bodies compared with wild type (187). Thus, ROS also have an immunity function.

One possible cause of the immunity defect in the *noxABC*^−^ mutant is the abrogation of DNA extracellular trap (ET) formation (Figure 1). The slug stage can persist for multiple days, during which it migrates through a dangerous melange of infectious bacteria and fungi that could decrease and/or prevent spore production. Phagocytic flux is limited in non-feeding developed cells (188). Protection from infection and intoxication appears to be mediated in part by a subpopulation of cells (<1%) that retain the capacity for phagocytosis (77). These so-called sentinel cells (S cells) are motile within the slug and phagocytose bacteria and toxins until they are eventually shed. Compared with the other cell types in slugs, S cells are enriched for *tirA* mRNA, and S cells from *tirA*^−^ mutants have a decreased capacity to kill bacteria (77). In response to bacteria or LPS, S cells secrete mitochondrially derived ETs *via* a mechanism that requires both TirA and NOX-generated ROS, and increased contamination of fruiting bodies correlates with decreased ET production (187). Binding of the TLR2 TIR domain with Nox2 during *M. tuberculosis* infection of macrophages has been reported (189). How NOX and TirA fit into the pathway and whether they interact awaits further examination, as do the questions of whether S cells kill intracellular bacteria and, if so, whether TirA and NOX are involved. ET formation during the growth phase of the *D. discoideum* life cycle has not been observed.

First discovered in neutrophils and named neutrophil extracellular traps (NETs), ETs have been observed in numerous immune cell types including macrophages (190–193). ETs comprise antimicrobial peptides, proteases, and signaling molecules bound to a meshwork of DNA released from the nucleus or mitochondria [(190); reviewed in Ref. (194)]. The mechanisms by which ETs kill extracellular bacteria and/or prevent their dissemination are not well understood. Neutrophils from CGD patients fail to generate NETs (195), although NOX-independent mechanisms have also been described (196–199), some of which utilize ROS from mitochondria (200). ET production by S cells further illustrates the conservation of cell-autonomous defense mechanisms in *D. discoideum*, a precursor to the specialized phagocytes of the vertebrate immune system (201).

### Divalent Metals

Maintaining the concentration of divalent trace metals such as iron, manganese, zinc, and copper is essential for every living organism to preserve metabolism and cell growth. Metalloproteins with trace metals as cofactors play a role in many important cellular functions such as signaling, respiration, transcription, translation, and cell division. Tight regulation of divalent metals is necessary: low levels of iron and manganese have detrimental metabolic effects, whereas zinc and copper are toxic at high concentrations.

For intracellular bacterial pathogens, trace metals are an important micronutrient resource with a role in many metabolic processes and are, as a consequence, essential for intracellular growth. Host cells such as macrophages have developed strategies to control growth of intracellular bacteria by sequestering metals such as iron and manganese (i.e., metal deprivation or nutritional immunity) or by pumping toxic metals inside the pathogen-containing compartment [i.e., metal poisoning; reviewed in Ref. (202–204)]. Bacteria have established ways to counteract metal deprivation or poisoning by expressing siderophores (i.e., small molecules with high affinity for the relevant metal) and uptake systems or by upregulating efflux systems such as P-type ATPases (203).

Consequently, the phagosomal concentration of essential trace metals varies during phagocytosis and infection with bacterial pathogens. Wagner et al. elegantly measured the metal concentration in the phagosome upon macrophage activation with inflammatory cytokines and upon infection with *M. tuberculosis* or *Mycobacterium avium* [(205); reviewed in Ref. (206)]. At 1-h post infection (hpi), the early phagosome was shown to be enriched in sulfur and chloride and depleted of calcium and potassium. At a later stage (24 hpi), the MCV harbored zinc, iron, and calcium at high concentrations. In addition, activation of infected macrophages with cytokines leads to a large increase in zinc and copper and a depletion of iron and chloride (205). Importantly, the metal concentration in the phagosome is very likely coupled to the proton gradient. Acidification of the phagosome is achieved by the combined actions of the V-ATPase and the Hv1 H^+^-channel (207). To counter-balance the electrogenic H^+^-gradient across the phagosomal membrane, Cl^−^ is imported into the phagosome by transporters of the CFTR (208) and CLC family [(209); reviewed in Ref. (210)], respectively [reviewed in Ref. (206)]. Lysosomal acidification is also facilitated by the efflux of cations (211).

#### Metal Poisoning

Zinc serves as a cofactor for more than 3,000 metalloproteins and is consequently the second most abundant trace element after iron. Zinc is redox neutral and has many roles in various biological processes as structural, catalytic and signaling component [reviewed in Ref. (212)]. It is essential for macrophage antimicrobial functions and controls among other processes monocyte chemotaxis, phagocytosis, and cytokine production [reviewed in Ref. (204)]. Intracellular zinc homeostasis is tightly regulated. 50% of zinc is present in the cytoplasm, whereas 30–40% can be found inside the nucleus and 10% is bound to membranes [reviewed in Ref. (212)]. To keep the cytosolic concentration of free zinc low (i.e., in the low nanomolar range), it is either bound to metalloproteins or metallothioneins or sequestered into membrane-bound organelles. Zinc is transported through biological membranes by various zinc transport proteins that are classified as zinc transporters (ZnTs, also cation diffusion facilitators) or Zrt-, Irt-related proteins (ZIPs) [reviewed in Ref. (212)]. Whereas ZnTs mediate the zinc transport from the cytosol to either organelles or the extracellular space, the ZIP family mediates transport into the cytosol from the extracellular space or organelles [reviewed in Ref. (212)]. Besides ZnT and ZIP transporters, various other proteins have been shown to mediate zinc transport, such as calcium channels (213), mucolipin-1 in interaction with TMEM163 (214), and members of the NRAMP family [reviewed in Ref. (215)].

Eleven putative zinc transporters have been previously identified in *D. discoideum* and categorized by functional analogy to mammalian zinc transporters into different subgroups (216). Three of the 11 were classified as members of a “ZIP subfamily” and 4 as members of an “LZT-like subfamily” (216). These two subfamilies correspond to the combined ZIP I and ZIP II subfamilies and the LIV-1 subfamily of mammalian ZIPs, respectively (212). Importantly, the members of the LZT-like family were named ZntA–ZntD even though they were classified as ZIP transporters, and four potential ZnT transporter homologs encoded in the *D. discoideum* genome were grouped by the authors as the “Cation efflux subfamily” (216).

**Figure 4:**
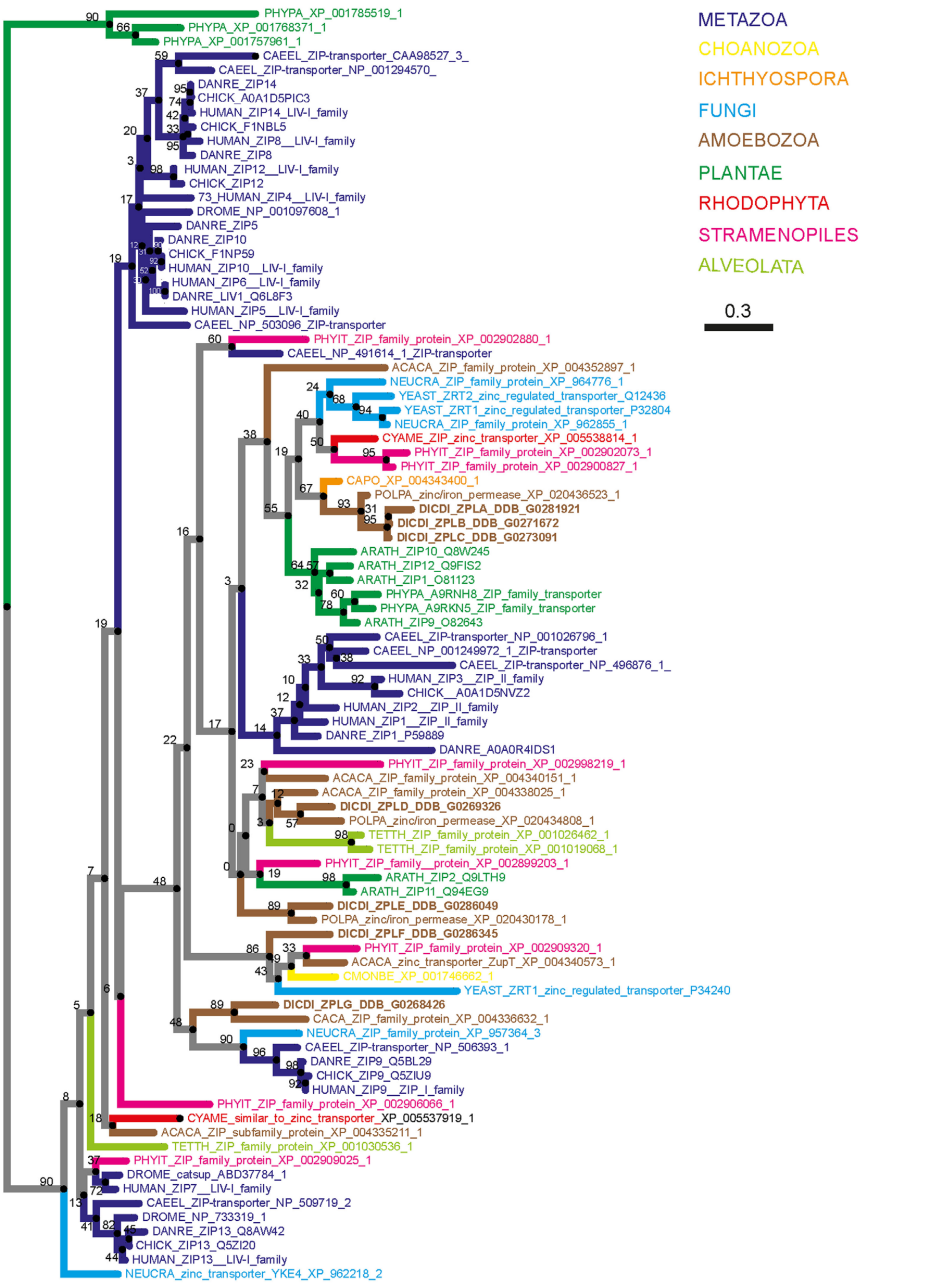
Phylogenetic tree of the Zrt-, Irt-related protein (ZIP) family. The sequence of human ZIP7 was used to search for zinc transporters of the ZIP family among non-redundant sequences in selected organisms using NCBI PSI-BLAST. A score of 2 × 10^−5^ was used as a threshold. Sequences were aligned with the software AliView [(218); http://ormbunkar.se/aliview/] and curated manually, to remove divergent N- and C-terminus. Trees were built using MAFFT and the E-INS-i strategy (219).

To demonstrate that the proteins initially named ZntA–ZntD belong to the ZIP family of zinc transporters and are not ZnTs, we generated two simplified phylogenetic trees comparing the sequences of various *D. discoideum* zinc transport proteins with zinc transporters of other taxonomic groups such as amoebozoa, fungi, plantae, and metazoa. These taxonomic groups were chosen based on previously published phylogenetic studies (176, 217). According to our phylogenetic tree of ZIP transporters (Figure 4) an unequivocal classification of the seven *D. discoideum* ZIP-like proteins (Zpl) into ZIP I, ZIP II, and LIV-1 subgroups by analogy to the human classification, as was done previously (216), is not possible. Three of the seven ZIP transporters are more similar to each other and cluster in one group (ZplA–C) that is more related to the ZIP transporters of fungi and ZIP II transporters of mammals. The three proteins ZplD–ZplF are more similar to proteins of Amoebozoa, Stramenopiles, and Plantae. ZplG is more related to the human ZIP I subfamily.

**Figure 5:**
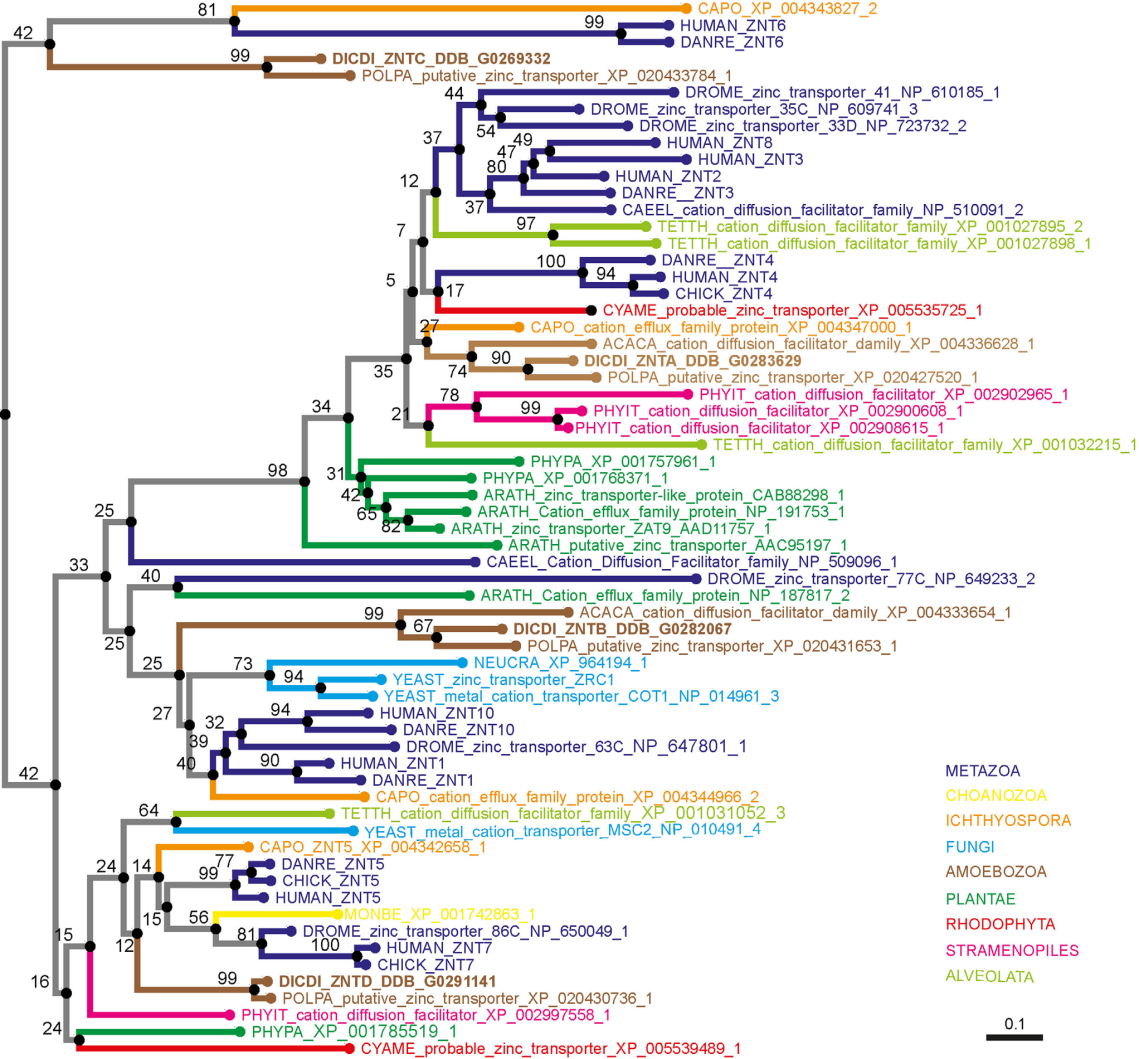
Phylogenetic tree of the ZnT family. The sequence of human ZNT4 was used to search for zinc transporters of the ZnT family among non-redundant sequences in selected organisms using NCBI PSI-BLAST. A score of 2 × 10^−5^ was used as a threshold. Sequences were aligned with the software AliView [(218); http://ormbunkar.se/aliview/] and curated manually, to remove divergent N- and C-terminus. Trees were built using MAFFT and the E-INS-i strategy (219).

Similarly, our phylogenetic tree of ZnT transporters clearly shows that the four proposed *D. discoideum* zinc transporters identified based on their homology to ZnTs indeed belong to this family (Figure 5). Consequently, we propose renaming the various transporters as outlined in Table 1 according to their respective family names (Zpl or ZnT). The *D. discoideum* proteins ZntC and ZntD are likely homologs of the human proteins ZNT6 and ZNT7, which are located in the early secretory pathway and contribute to the activation of zinc-containing enzymes (220). Whereas ZntA does not have a close mammalian homolog, the closest human relatives of ZntB are the early endosomal protein ZNT10 and the plasma membrane protein ZNT1 (212).

**Table 1:** Proposed nomenclature of zinc transporters in *Dictyostelium discoideum*.

Gene ID	Gene product	Proposed name
DDB_G0281921	Zrt-, Irt-related protein (ZIP) zinc transporter protein, zinc/iron permease	zplA (ZIP-like A)
DDB_G0271672	ZIP zinc transporter protein, zinc/iron permease	zplB
DDB_G0273091	ZIP zinc transporter protein, zinc/iron permease (there is a gene duplication in AX3 and AX4, but not in AX2)	zplC1 and 2
DDB_G0269326 (previously *zntD*)	Zinc/iron permease, zinc transporter	zplD
DDB_G0286049 (previously *zntC*)	Zinc/iron permease, zinc transporter	zplE
DDB_G0286345 (previously *zntB*)	Zinc/iron permease, zinc transporter	zplF
DDB_G0268426 (previously *zntA)*	Zinc/iron permease, zinc transporter	zplG
DDB_G0283629	Putative zinc transporter, cation diffusion facilitator (CDF) family protein	zntA (zinc transporter A)
DDB_G0282067	Putative zinc transporter, CDF family protein	zntB
DDB_G0269332	Putative zinc transporter, CDF family protein	zntC
DDB_G0291141	Putative zinc transporter, CDF family protein	zntD

At the host–pathogen interface, zinc deprivation (221) or zinc poisoning are strategies of mammalian professional phagocytes to restrict intracellular bacteria growth. Both processes are probably highly dependent on zinc transporter proteins (222, 223) During *M. tuberculosis* infection, it was proposed that free zinc is released from metallothioneins *via* the oxidative burst that is induced upon infection and, consequently, zinc poisoning was proposed as “a new chapter in the host–microbe arms race” (224). The contributions of zinc transporters to cell-autonomous defenses of macrophages and *D. discoideum* await elucidation.

In contrast to zinc, copper is redox active and cycles under physiological conditions between the two oxidative states Cu^+^ (i.e., cuprous) and Cu^2+^ (i.e., cupric). Consequently, copper serves as an ideal cofactor for electron transfer and redox reactions such as respiration and detoxification of free radicals [reviewed in Ref. (225)]. Proteins involved in copper uptake, sequestration, and trafficking regulate copper homeostasis in eukaryotic cells [reviewed in Ref. (226)]. Copper uptake into the cytosol is mediated by the copper permease CTR1 [reviewed in Ref. (227)]. Similar to zinc, copper belongs to the so-called “death metals” and is toxic at high concentrations. Therefore, by analogy to zinc, low copper concentrations in the cytosol are maintained by metallothioneins. Directly after its uptake, chaperones such as ATOX1, CCS, and COX17 are involved in copper trafficking inside the cytoplasm (228).

Copper is imported into the *trans*-Golgi network by the action of two P-type ATPases: ATP7A and ATP7B. ATP7A is also located at the plasma membrane, where it mediates copper efflux from the cytosol to the extracellular space, and at the phagosomal membrane, where it imports copper from the cytosol into the phagosomal lumen [reviewed in Ref. (226, 228)].

Copper has many antimicrobial properties. Its ability to switch between two oxidation states supports the production of hydroxyl radicals *via* the Fenton- and the Haber–Weiss reactions [reviewed in Ref. (229)]. In addition, copper is able to disrupt the structure of proteins, and Cu^2+^ might be able to disrupt Fe–S clusters. In macrophages, copper transport proteins such as ATP7A that mediate Cu^2+^ import into the phagosome are induced upon infection and stimulation with inflammatory cytokines (230). Bacteria have evolved strategies to overcome high copper concentrations. For instance, a multi-copper oxidase is required for copper resistance of *M. tuberculosis*, probably by oxidizing toxic Cu^2+^ in the periplasm (231).

The *D. discoideum* genome encodes one Ctr-type copper permease (i.e., p80) that, by analogy to mammalian cells, should mediate copper uptake into the cytosol, and three putative copper-translocating P-type ATPases that were annotated as *atp1* (DDB_G0273675), *atp2*, the ortholog of ATP7A (DDB_G0284141), and *atp3* (DDB_G0269590) (232, 233). ATP1 was induced upon incubation of *D. discoideum* with copper salts, leading to the conclusion that ATP1 is responsible for copper tolerance in *D. discoideum* (233). Expression of ATP7A and ATP3 was increased upon bacteria ingestion and decreased when bacteria and copper salts were added together, arguing for a possible role in copper trafficking during phagocytosis and killing of bacteria. The increased expression of p80 only upon incubation with bacteria suggests that copper is needed for bacterial killing (233).

#### Nutritional Immunity

In contrary to metal poisoning, nutritional immunity does not kill the bacteria but restricts its intracellular growth. During infection, pathogens need to acquire essential nutrients from the host such as amino acids, lipids, sugars, and, importantly, transition metals. Thus, to restrict their availability to the pathogen, these metals are depleted from the phagosome. The best studied metals that are sequestered by the host are iron and manganese (206, 234–236).

Nutritional immunity of transition metals is controlled in part by the Natural Resistance-Associated Macrophage Protein (i.e., NRAMP) family of divalent-metal transmembrane transporters (237–239). The NRAMP family is widely represented from bacteria to mammals (240, 241), as well as in plants (242) and yeast (243). The family members play an important role in intracellular metal-ion homeostasis and are able to transport a broad range of transition metals (215, 244).

In mammals, two NRAMP members have been identified: NRAMP1 [(SLC11A1) (237)] and NRAMP2 [(SLC11A2, DMT1, or DCT1) (241)]. Between these proteins, 63% of residues are identical and 15% are highly conservative substitutions, and they share very similar secondary structures with hydrophobic cores of 10 transmembrane segments (245). Regarding divalent-metal affinity, NRAMP1 has a clear preference for Mn^2+^, Fe^2+^, and Co^2+^ (246), whereas NRAMP2 transports those three metals and also Zn^2+^, Cd^2+^, Cu^2+^, Ni^2+^, and Pb^2+^ (241). NRAMP1 expression is restricted to late endocytic compartments (i.e., endo-lysosomes) of professional phagocytes such as macrophages and neutrophils (247). NRAMP2 is ubiquitously expressed in all mammalian cells and is located at the plasma membrane (248). In addition, NRAMP2 was observed at the apical membrane of enterocytes as well in recycling endosomes (249). Mutations in *nramp2* lead to severe microcytic anemia related to an iron absorption deficiency (250, 251). Both NRAMP1 and NRAMP2 might be involved in neurodegenerative diseases such as Parkinson’s (252, 253). NRAMP1 contributes to the resistance to intracellular bacterial infection. Indeed, depletion of NRAMP1 leads to an increased susceptibility of mice to several intracellular pathogens such as *Mycobacterium* species, *Leishmania donovani*, and *Salmonella* species (237, 254–259) by impairing phagosomal acidification and reducing fusion with the lysosomes (260). In human, *nramp1* polymorphic variants are associated with susceptibility to tuberculosis (261, 262) or leprosy (263).

Although it is accepted that NRAMPs depend on a V-ATPase-generated proton gradient to drive metal transport (264), the direction of metal transport at the phagosomal membrane remains controversial. Whether NRAMP1 is an antiporter or a symporter and whether NRAMP1 imports or depletes metals from the phagosome are unclear. On the one hand, some authors suggest that NRAMP1 acts as an antiporter of protons and delivers cation metals into the phagosome, contributing to the generation and accumulation of toxic free radicals involved in bacteria killing (265–268). On the other hand, the hypothesis that NRAMP1 operates as a symporter of protons to efflux metals from the phagosome to the cytosol is better supported by the literature and is consistent with the function of its paralog NRAMP2. This is in line with the current hypothesis of nutritional immunity in which pathogen access to metals is restricted (246, 269). These two different scenarios are nicely described in previous reviews (215, 244, 270).

**Figure 6:**
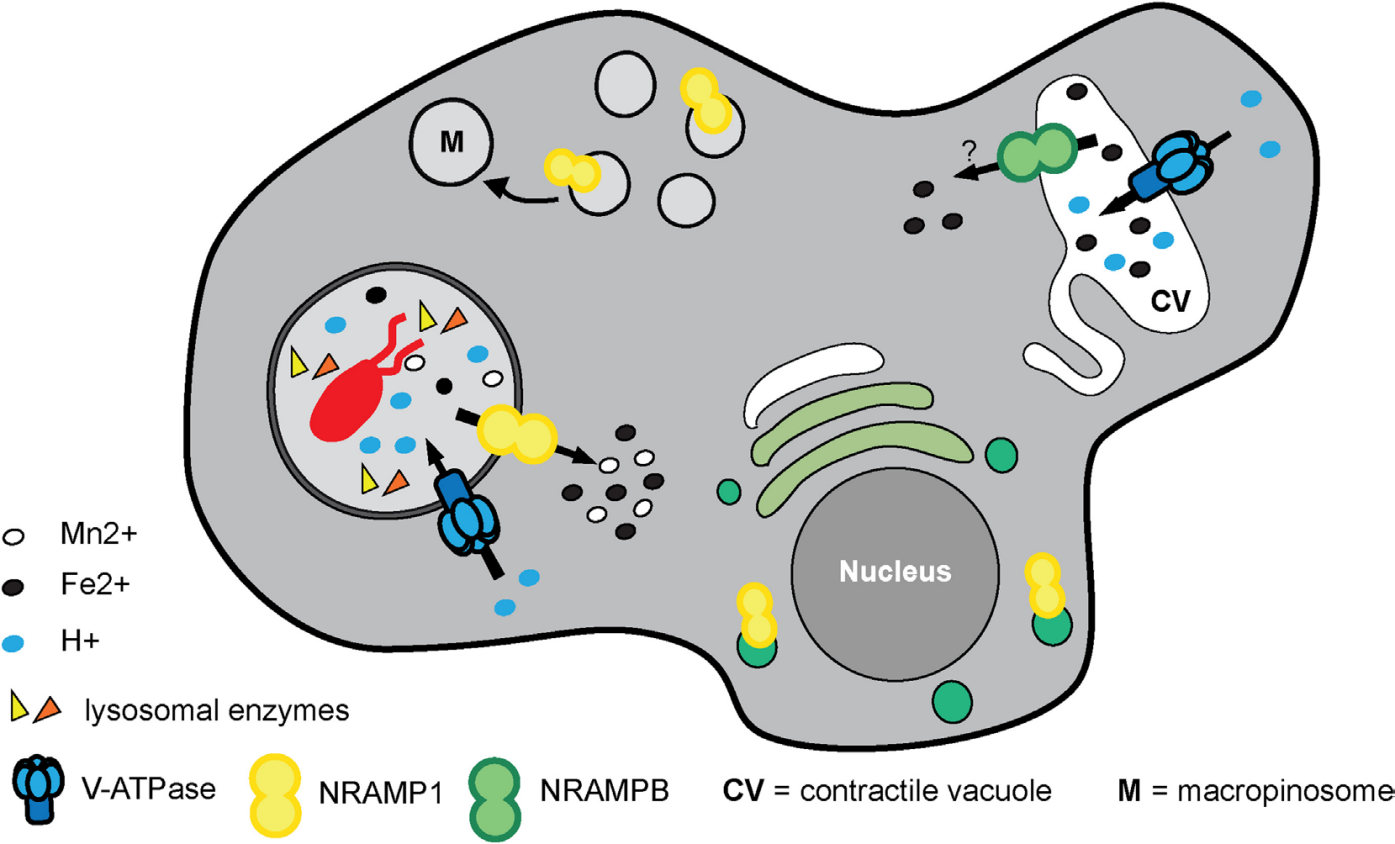
“Nutritional immunity” and homeostasis of transitional metals orchestrated by NRAMP transporters in *Dictyostelium discoideum*. NRAMP1 is localized at macropinosomes, phagosomes, and the Golgi network whereas NRAMPB is exclusively found in the membrane of the contractile vacuole. Both co-localize with the vacuolar ATPase (V-ATPase), but only NRAMP1 is dependent on the H^+^-gradient to efflux metals from the phagosome to the cytosol to restrict metal availability to the pathogen in the process referred to as “nutritional immunity.” NRAMPB, together with NRAMP1, contributes to iron homeostasis and regulates osmolarity inside the cell independent of the H^+^-gradient. Although the literature supports it as similar to the symporter NRAMP1, its role as symporter or antiporter still remains to be clearly defined.

The *D. discoideum* genome encodes two NRAMP proteins called NRAMP1 (DDB_G0276973) and NRAMPB (DDB_G0275815, formerly NRAMP2). NRAMP1 is an archetypical NRAMP protein, orthologous to NRAMP1 in mammals, whereas NRAMPB is not the ortholog of NRAMP2 in mammals but rather is more closely related to the prototypical NRAMP from bacteria (271). Both transporters are in different subcellular compartments; however, they both co-localize with the V-ATPase. NRAMP1 localizes to macropinosomes and phagosomes with the V-ATPase, and NRAMP1 also localizes to the Golgi region (272). NRAMPB is exclusively found in the membrane of the contractile vacuole (CV) (273), which is enriched for the V-ATPase (108, 274) but has a neutral pH. Single *nramp1* or *B* null mutants exhibit slower growth than wild type under conditions of iron depletion while a double *nramp1* and *B* mutant, but not single mutants, is more resistant than wild type to iron overload (273). These results suggest that NRAMPB and NRAMP1 act non-redundantly to regulate iron homeostasis and that the CV serves as a transient storage compartment for metal cations (Figure 6). During infection, an *nramp1* mutant strain is more permissive for intracellular growth of *Mycobacterium* species and *L. pneumophila* (272), and *nrampB* null or *nramp1* and *B* double null mutants are more permissive for *L. pneumophila* growth (effects of *nrampB* deletion on the growth of *Mycobacterium* species have not been reported) (273). Moreover, *L. pneumophila* inhibits the recruitment of the V-ATPase, which attenuates the antimicrobial effects of NRAMP1 by preventing its proton-driven iron transport activity (46). A recent study demonstrates that, in addition to having an impact on phagosomal iron concentration, both NRAMP1 and NRAMPB influence the translocation efficiency of *F. noatunensis* from the bacteria-containing compartment into the cytosol, possibly due to alterations in phagosome maturation (53). This new insight into NRAMP function is in line with the results obtained for *M. tuberculosis* infection in macrophages, in which *nramp1* deletion induced a higher level of escape from its vacuole (275).

As described earlier, the directionality of the metal transport mediated by NRAMP1 is still poorly understood. Studies in *D. discoideum* suggest transport into the cytosol. In assays with purified phagosomes, iron export was NRAMP1- and ATP dependent (272). This observation is consistent with NRAMP1 acting as a symporter that uses a V-ATPase-generated proton gradient to transport iron out of the phagosome. Using the iron-chelating fluorochrome calcein, it was shown that NRAMP1 mediates iron efflux from macropinosomes *in vivo* (271). In addition, to obtain better insight into the ion selectivity of NRAMP1, the authors used *Xenopus* oocytes expressing NRAMP1, NRAMPB, or rat DMT1/NRAMP2 (used as an internal control). Interestingly, it was shown that NRAMP1 and DMT1 are able to transport Fe^2+^ and Mn^2+^ but not Fe^3+^ or Cu^2+^ in an electrogenic and proton-dependent manner, whereas NRAMPB transports only Fe^2+^, and this in a non-electrogenic manner independently from protons (271) (Figure 6).

## Autophagy

As described earlier, phagocytosis is the major mechanism by which *D. discoideum* digests intracellular bacteria with the purpose of nutrient acquisition. However, pathogenic microbes have evolved mechanisms to escape degradation within phagosomes (276). In *D. discoideum*, as in other eukaryotic phagocytes, bacterial escape from the phagosome triggers a more stringent catabolic pathway named autophagy, which serves as an additional defense mechanism for the infected amoeba [a comprehensive review on autophagy in *D. discoideum* can be found in Ref. (277)]. The autophagic process by which intracellular pathogens and/or their damaged phagosomes are specifically recognized and digested is termed xenophagy.

**Figure 7:**
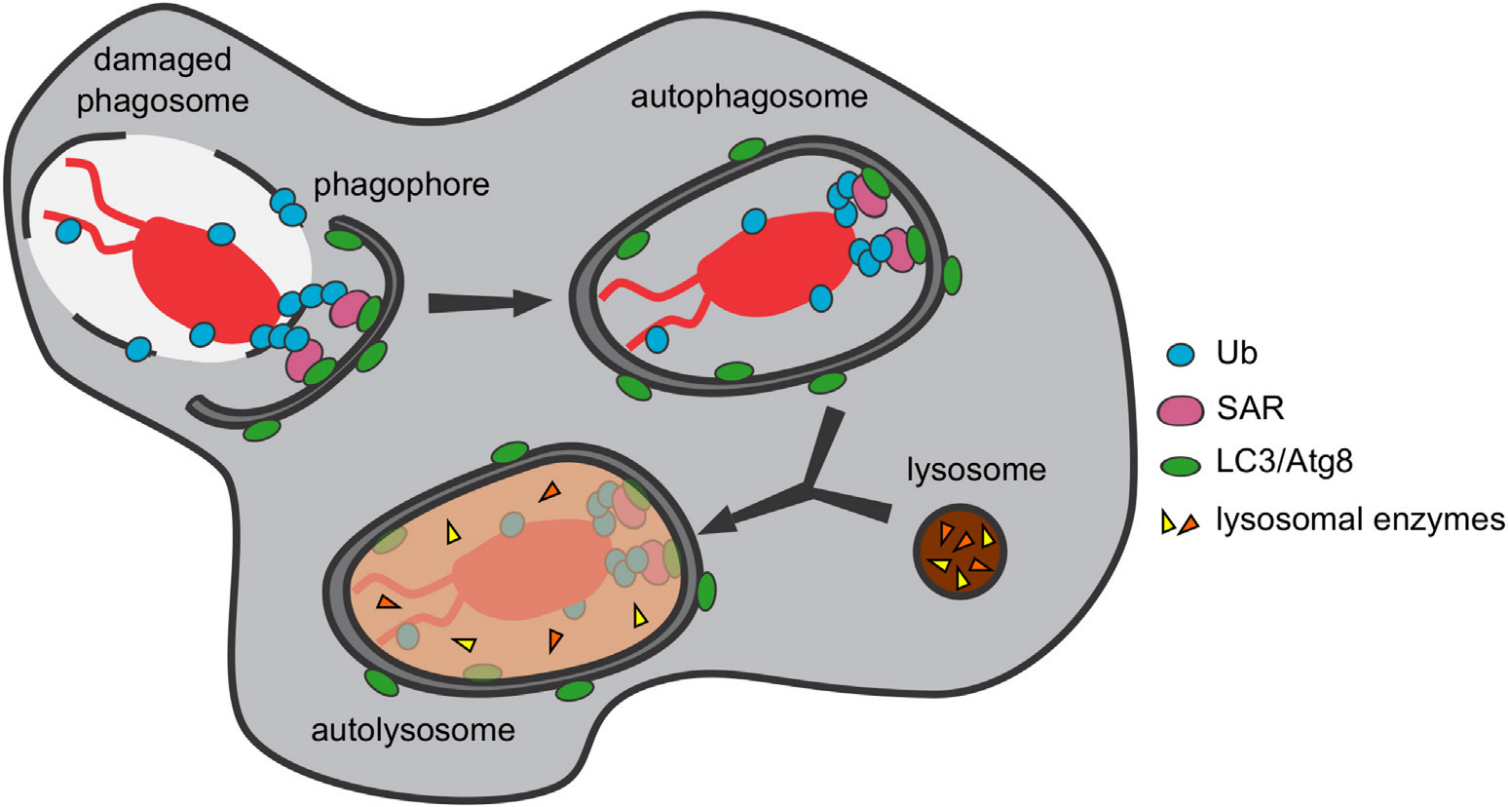
Pathogenic bacteria are captured and digested by xenophagy. The autophagic machinery of *Dictyostelium discoideum* recognizes and captures cytosolic bacteria and/or their damaged phagosomes in autophagosomes, where they are digested upon fusion with lysosomes.

The autophagy pathway consists of the formation, upon induction by various stresses such as oxidation, nutrient starvation, or microbial infection, of a double-membrane cisterna, the phagophore, at multiple sites on the ER (36). During xenophagy, the membranes of the phagophore expand around the cytosolic bacterium and/or its damaged compartment to finally engulf them in a closed vacuole called the autophagosome, which, upon fusion with lysosomes, forms an acidic and degradative compartment, the autolysosome, where bacterium and membranes are digested (Figure 7). Many of the proteins involved in the process of autophagosome formation (for instance, proteins forming part of the TORC1, the ULK/Atg1 and the phosphatidylinositol 3-kinase complexes) are conserved between mammalian cells and *D. discoideum* (278). Interestingly, certain autophagy proteins conserved in both humans and *D. discoideum* are actually absent in *Saccharomyces cerevisiae*, making their study in this amoeba a perfect complement to those already performed in yeasts (279).

Specific receptor proteins are in charge of recruiting the phagophore membranes to the bacterial cargos or the remnants of the bacteria-containing compartment, which are tagged with ubiquitin for degradation. These receptors contain a ubiquitin-binding domain, which recognizes the ubiquitinated material, and an LC3-interacting region, which binds the phagophore membrane through interaction with LC3/Atg8, the main autophagosomal marker (280). In *D. discoideum*, the only selective autophagy receptor identified so far is p62/SQSTM1 (278), which has been shown to recognize the intracellular pathogens *F. noatunensis* and *M. marinum* (51, 52, 130). In addition, the mRNA levels of *p62/sqstm1* increase upon infection of *D. discoideum* with both bacterial pathogens, which, in the case of *M. marinum*, has been demonstrated to be dependent on the membrane damage caused by the bacterium. However, *M. marinum* avoids its xenophagic killing by presumably blocking lysosomal fusion (51), a mechanism of intracellular mycobacterial survival that was previously proposed to occur in human dendritic cells during infection with *M. tuberculosis* (281). Other bacteria known to be captured and digested by xenophagy in this amoeba are *S. enterica* and *S. aureus* (54, 282).

In addition to pathogenic bacteria, *D. discoideum* xenophagy also fights against various *S. cerevisiae* strains (283). Mutant amoebae lacking the autophagy proteins Atg5, Atg6, Atg7, or Atg8 have a decreased capability of preying on this fungus. However, in *atg1*- amoebae, which cannot produce autophagosomes, *S. cerevisiae, Candida albicans*, and *Candida glabrata* are surprisingly killed more efficiently. Koller and collaborators suggest, among other hypotheses, that the autophagic machinery might be used by these yeasts to escape *D. discoideum* in a non-lytic manner, as already shown for *M. marinum*. During ejection from the *D. discoideum* cytosol, the wound generated by the egress of this bacterium through the plasma membrane is sealed with phagophores (130). One might speculate that the yeasts could egress from *D. discoideum* by autophagosome exocytosis, a process already shown to occur in this amoeba during secretion of the spore differentiation factor 2 precursor AcbA (284). During this unconventional exocytosis, yeasts would be engulfed by autophagic membranes, which would then fuse with multivesicular endosomes before fusing with the plasma membrane to release the yeast. Amoebae lacking Atg1 might be unable to exocytose the yeast efficiently, thus trapping them and facilitating their death. Further investigations are required to validate this hypothesis.

## Conserved Microbial Restriction Factors: Lessons from MPS Cells

**Figure 8:**
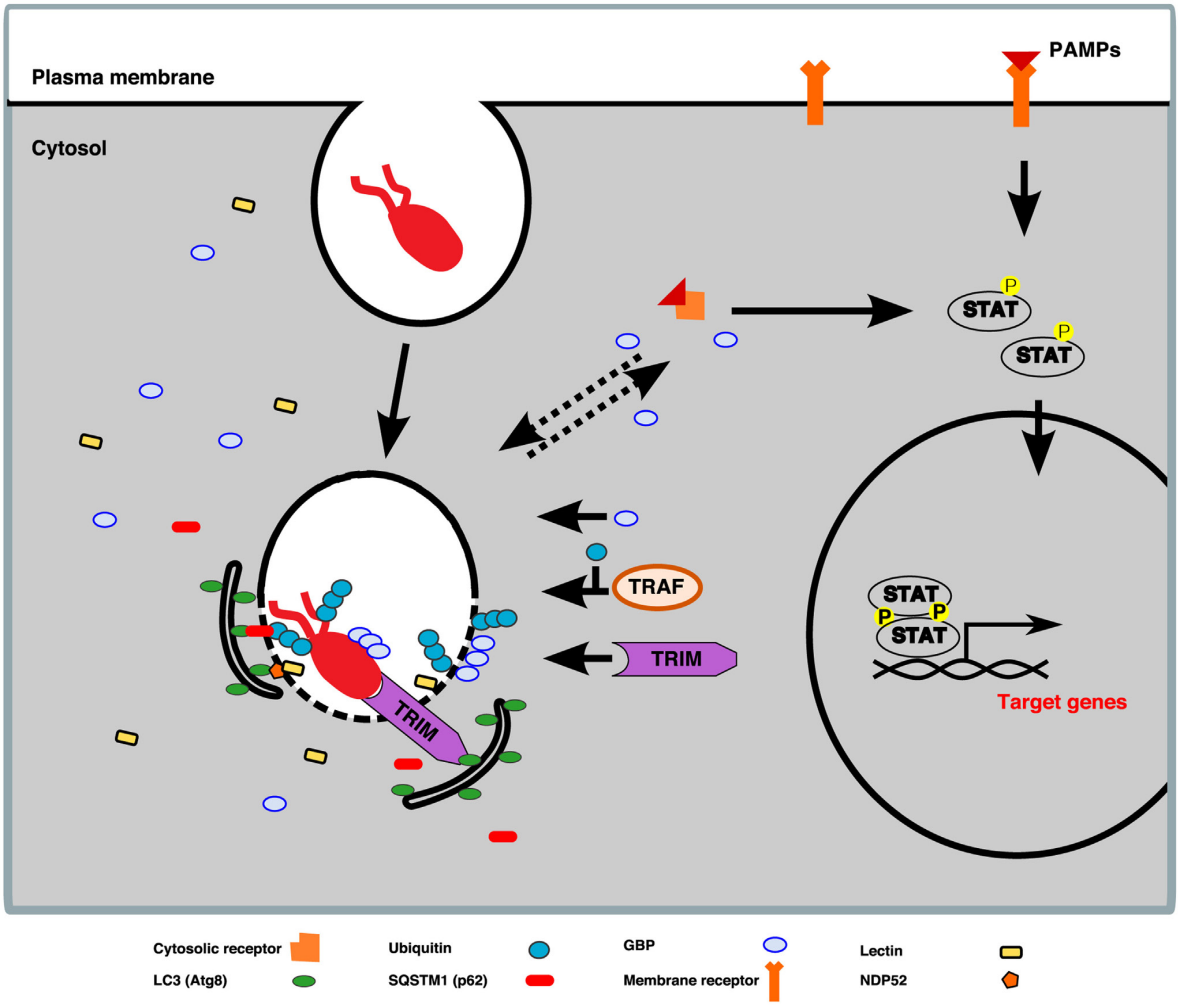
Working model of TNF receptor-associated factor (TRAF), tripartite motif-containing protein (TRIM), guanylate-binding protein (GBP), and signal transducers and activators of transcription (STAT) proteins in *Dictyostelium discoideum*. Lessons learned from studies in macrophages allow us to envision a model according to which, upon bacterium uptake by *D. discoideum*, pathogen-associated molecular patterns (PAMPs), or danger-associated molecular patterns, shared by a broad range of microbes, are detected by membrane or cytosolic receptors. The perception of pathogens leads to the activation of transcription factors from the STAT family, which are translocated to the nucleus, where they bind the promoters of innate-immunity-related target genes encoding galectins, GBPs, NADPH oxidases, SQSTM1, TRIMs, and NDP52. In addition, membrane damage of the pathogen-containing vacuole (PCV) exposes pathogens to the cytosol and permits their decoration with K63-linked polyubiquitin chains deposited by members of the TRAF E3-ligase family. The K63-linked polyubiquitination chains serve as a cue for recruitment of the autophagy machinery *via* autophagy cargo receptors, for instance, SQSTM1 (p62 in *D. discoideum*). Moreover, K63 ubiquitin-tagged membranes also promote the recruitment of GBP oligomers to the pathogen surface and/or PCV membrane, which facilitates bacteria killing and clearance. Furthermore, members of the TRIM E3-ligase family are able to detect and bind directly to the invading pathogen and mediate its degradation by autophagy. The aforementioned factors are likely to function in an interrelated manner in human macrophages, and it remains to be explored whether the *D. discoideum* versions are involved in its cell-autonomous defenses.

Studies conducted in MPS cells have identified numerous factors that are involved in the successful restriction of intracellular pathogens. Among these proteins are the glycan-binding galectins, TNF receptor-associated factors (TRAFs), and tripartite motif-containing proteins (TRIMs), which are E3 ubiquitin ligases, the guanylate-binding proteins (GBPs), a family of cytokine-induced large GTPases, and finally the signal transducers and activators of transcription (STAT) proteins. *D. discoideum* expresses a family of lectins, the discoidins, that might function analogously to galectins. Its genome also encodes homologs of TRAFs, TRIMs, GBPs, and STATs. Based on their counterparts in MPS cells, the *D. discoideum* versions of these restriction factors are likely to have immune functions (Figure 8).

### Discoidins

The galectins compose a family of 15 mammalian lectins with affinity for β-galactoside sugars that share a characteristic carbohydrate recognition domain (285). They have been classified into three groups according to their overall structure: the “prototype,” the “tandem-repeat,” and the “chimera-type.” Galectins are present in the cytosol (286) and nucleus (287) of cells but are also secreted extracellularly *via* an unconventional secretion mechanism that has remained elusive for decades (288). Galectins play a role in multiple biological processes, including angiogenesis (289), tumor growth (289), and inflammation (290), due to their ubiquitous localization as well as the high diversity of self and non-self glycoconjugates that they recognize [for a review, see Ref. (291)]. Importantly, extracellular lectins bind to the complex glycocalyx coat at the cell surface, which enables the formation of specific microdomains known as the galectin lattice (292). The galectin lattice restricts the mobility of glycoconjugates in the plasma membrane and has important functions in signaling and endocytosis (293). Some galectins recognize the surface of various pathogens (294–297) and have been proposed to have a direct antibacterial effect (298, 299). These interactions involve galectin binding to specific bacterial species or clades according to the carbohydrates displayed on their cell wall or capsules. In addition to their roles in the extracellular milieu, galectins have recently emerged as general innate-immune factors against a wide range of intracellular infections. This newly described function appears not to involve the binding of cytosolic galectins directly to the surface of intracellular pathogens but rather to result from the recognition of self glycans present on the luminal leaflet of pathogen-containing vacuoles. These glycans become accessible to the cytosolic lectins when membrane damage occurs, as shown during infection with *S. enterica, L. pneumophila*, and *Yersinia pseudotuberculosis* (300, 301). This process leads to the recruitment of the autophagy machinery (300) or of GBPs (301) to the compartment.

Although *D. discoideum* lacks galectin homologs, discoidins share molecular and biological characteristics with galectins. They form a family of four β-galactoside-binding and β-*N*-acetylgalactosamine-binding lectins with two carbohydrate binding domains (302, 303), and recent genome analyses identified three potential discoidin-like proteins in *D. discoideum* (http://dictybase.org). Discoidins are expressed throughout the *D. discoideum* life cycle (69, 70). They are highly abundant in the cytosol and are also secreted despite their lack of a signal peptide (304). Early reports suggested a possible role in self-recognition during the multicellular cycle of *D. discoideum* (305), but it was later observed that mutants with much reduced expression of discoidins (1–2% of wild type) were able to form apparently normal fruiting bodies (306). In addition, subsequent studies were unable to confirm surface localization of discoidins (307, 308). Consequently, the role of discoidins in self-recognition and adhesion during *D. discoideum* development has remained controversial for several decades. Whether discoidins bind bacteria and/or have a role in cell-autonomous defenses similar to that of galectins is currently under investigation.

### Ubiquitination

The significance of ubiquitination in the regulation of various aspects of mammalian immunity has been increasingly recognized in recent years. Ubiquitination is an omnipresent posttranslational modification in which the 76-amino acid ubiquitin is covalently linked to lysine (K) residues of substrate proteins. The stepwise enzymatic cascade of ubiquitination involves following three proteins: ubiquitin-activating enzyme (E1), ubiquitin-conjugating enzyme (E2), and ubiquitin ligase (E3). Their activity results in the attachment of one ubiquitin to the substrate protein linked by an isopeptide bond between the ubiquitin C-terminus and the NH_2_ group of the substrate K residue. This is referred to as monoubiquitination. Repeated ubiquitination leads to the generation of a polyubiquitin chain, known also as polyubiquitination. Ubiquitin contains seven K residues (K6, K11, K27, K29, K33, K48, and K63). Typically, the attachment of K48-linked polyubiquitin chains to substrate proteins serves as a signal for their degradation by the proteasome. However, the other linkages and the C- or N-terminal linear linkage of ubiquitin moieties play roles in almost all aspects of plant and animal biology, such as growth, development, stress responses, and immunity. In mammals, K63-linked polyubiquitination has been associated with a broad range of immunity-related processes and particularly the activation of the NF-κB pathway, xenophagy and apoptosis (309). *D. discoideum* has 13 genes encoding ubiquitin, and the TRAFs and TRIMs responsible for K63-linked polyubiquitination are conserved from *D. discoideum* to mammals.

### TNF Receptor-Associated Factors

TNF receptor-associated factors are a family of proteins primarily involved in the regulation of inflammation, antiviral responses, and apoptosis (309, 310). Currently, seven TRAF proteins have been characterized in humans: TRAF1–7. Typically, the TRAF proteins comprise an N-terminal RING domain that mediates the interaction between an E2 ligase and the substrate, followed by a zinc-finger domain, which may play a role in DNA, RNA, protein, and/or lipid binding, and a C-terminal TRAF homology (MATH) domain. The TRAF/MATH domain has an N-terminal TRAF region that mediates homo- and hetero-oligomerization between TRAF members and a C-terminal region that is important for interactions with receptors and adaptor proteins (310).

TNF receptor-associated factor 6, perhaps the most ancient mammalian TRAF, is a RING-type E3-ligase that ubiquitinates *via* the K63-linkage (311–313). It is required for mTORC1 translocation to the lysosome, and TRAF6-catalyzed K63 polyubiquitination modulates mTORC1 amino acid sensing capacity (314). Moreover, in macrophages, TRAF6 is responsible for the decoration of pathogens and pathogen-containing vacuoles with polyubiquitin chains, which serve as a cue for GBP recruitment and as a recognition signal for the autophagy cargo receptor p62 (313, 315).

**Figure 9:**
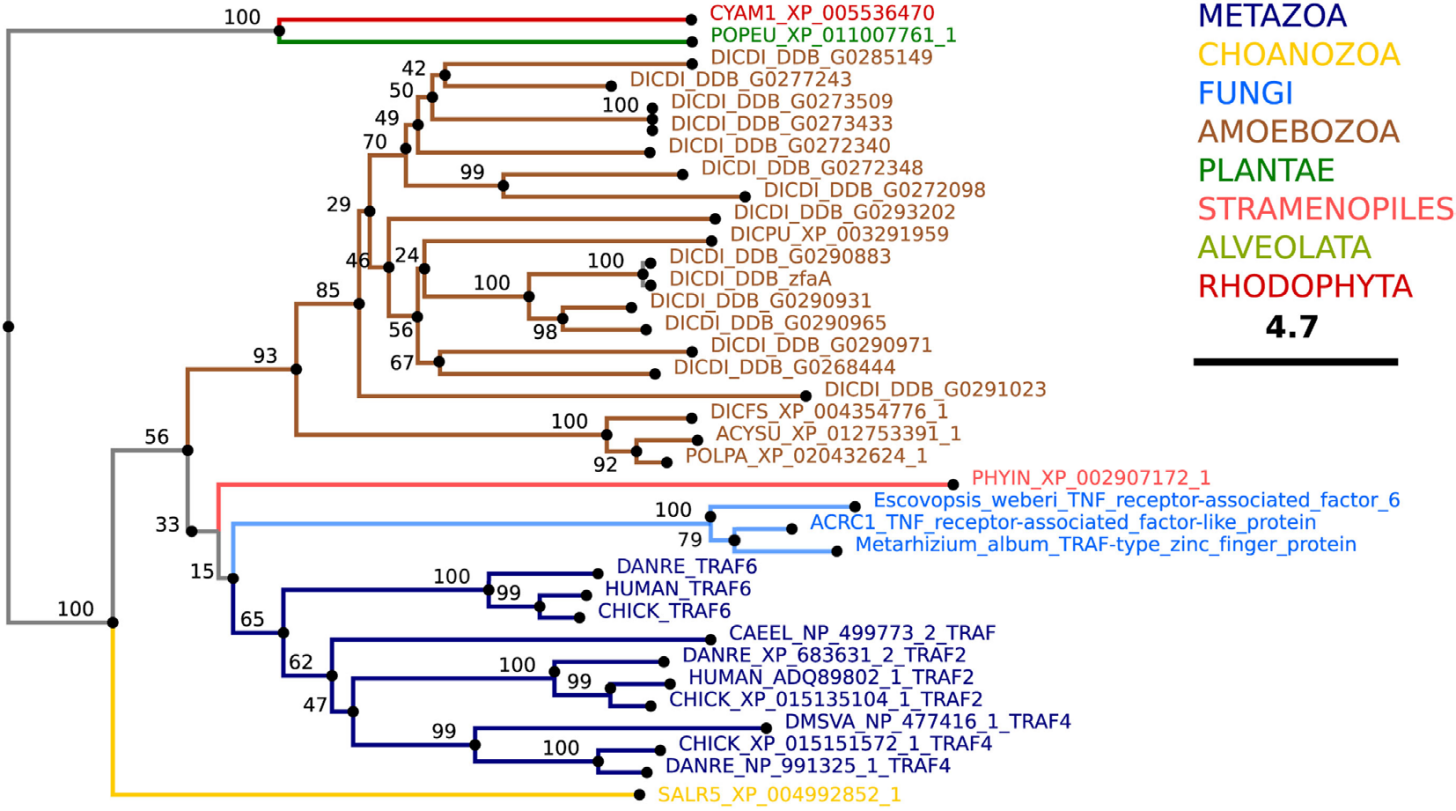
Theoretical phylogenetic relations of TNF receptor-associated factor (TRAF) proteins. The sequence of human TRAF6 was used to search for TRAF homologs among the non-redundant sequences in selected organisms using NCBI PSI-BLAST. A score of 2 × 10^−5^ was used as a threshold. Similarities of the selected sequences were determined using BLOSUM62 matrix and E-INS-i strategy (219). Sequences were manually curated using AliView software [(218); http://ormbunkar.se/aliview/], and the resulting final alignment was used to generate a neighbor joining phylogenetic tree (NJ, bootstrap 1,000×).

BLAST analyses predict that TRAF-like proteins are also present in several social amoeba species. More than 40 TRAF-like proteins in *D. discoideum* are predicted, of which 16 contain the RING, zinc-finger and TRAF domains present in mammalian TRAF2, TRAF3, TRAF5, and TRAF6 (Figure 9; Table 2). Despite the fact that human and *D. discoideum* TRAF proteins show significant similarities with respect to their major domains, their evolutionary divergence precludes ortholog assignment. Determining whether *D. discoideum* TRAFs regulate nutrient sensing and/or ubiquitinate pathogens will expand our understanding of their roles in infection.

**Table 2:** Pairwise comparisons of the human TNF receptor-associated factor (TRAF) 6, tripartite motif-containing protein (TRIM) 37, guanylate-binding protein (GBP) 3, and signal transducers and activators of transcription (STAT) 2 with their putative *Dictyostelium discoideum* homologs.

*D. discoideum*	*Homo sapiens*	Bit score	*e*-Value	Identity (%)	Proposed name
dstD	STAT2	57.4	4e−12	26	NA
dstA	STAT2	77.4	4e−12	29	NA
dstC	STAT2	56.2	2e−11	25	NA
dstB	STAT2	71.6	1e−09	28	NA
DDB_G0281639	GBP3	137	3e−38	26	DdGBP
DDB_G0272454	TRAF6	152	1e−15	22	DdTRAFa
DDB_G0285149	TRAF6	79.7	1e−19	20	DdTRAFb
DDB_G0290883	TRAF6	70.1	2e−16	22	DdTRAFc
DDB_G0290961	TRAF6	75.9	2e−18	21	DdTRAFd
DDB_G0290971	TRAF6	73.5	9e−12	26	DdTRAFe
DDB_G0277243	TRAF6	84.7	1e−20	21	DdTRAFf
DDB_G0272340	TRAF6	80.1	4e−19	21	DdTRAFg
DDB_G0293202	TRAF6	72.0	1e−16	23	DdTRAFh
DDB_G0272348	TRAF6	92.8	1e−15	23	DdTRAFi
DDB_G0273433	TRAF6	68.6	2e−15	23	DdTRAFj
DDB_G0273509	TRAF6	68.6	2e−15	23	DdTRAFk
DDB_G0290931	TRAF6	97.0	2e−15	23	DdTRAFl
DDB_G0290965	TRAF6	65.5	2e−14	25	DdTRAFm
DDB_G0268444	TRAF6	61.6	3e−13	20	DdTRAFn
DDB_G0291023	TRAF6	79.7	2e−10	26	DdTRAFo
DDB_G0272098	TRAF6	70.1	7e−10	22	DdTRAFp
DDB_G0273381	TRIM37	188	1e−46	28	DdTRIM

*The BLOSUM62 pairwise alignment was performed with NCBI BLASTp suite-2 sequences. This table shows the similarity scores with these D. discoideum homologs and their proposed name*.

### Tripartite Motif-Containing Proteins

The TRIM superfamily is remarkably conserved among metazoans and, perhaps as a result of an expansion during vertebrate evolution, is represented by more than 80 members in humans. TRIMs typically comprise an N-terminal RING domain, a B-box domain containing several zinc-binding motifs, a coiled-coil domain, and a considerably diverse C-terminal domain important for substrate binding (316).

Tripartite motif-containing proteins are important for many aspects of immunity resistance to pathogens. Recent studies in mouse macrophages demonstrate that various TRIM proteins are induced upon infection with influenza virus or activation of TLRs in a type-I-interferon (IFN)-dependent manner (317, 318). TRIMs are involved in restriction of HIV replication and activation of NF-κB downstream of TLRs (318). Moreover, they play a dual role as receptors and regulators of autophagy. As regulators, TRIMs serve as platforms for the assembly of the core autophagy initiators ULK1 (Atg1 in yeast and *D. discoideum*) and Beclin1 (Atg6 in yeast and *D. discoideum*) (319). In macrophages, autophagy cargo receptors recognize and bind K63-linked polyubiquitin chains and galectins, which serve as “eat-me” signals and mediate the binding of the cargo to phagophore-conjugated LC3. As receptors, TRIMs are able to recognize endogenous and exogenous (e.g., bacteria) cargo intended for autoloysosomes *via* binding of their diverse C-terminal domains to the cargo in a ubiquitin-independent manner and mediate delivery to the phagophore by also binding LC3 (320, 321).

**Figure 10:**
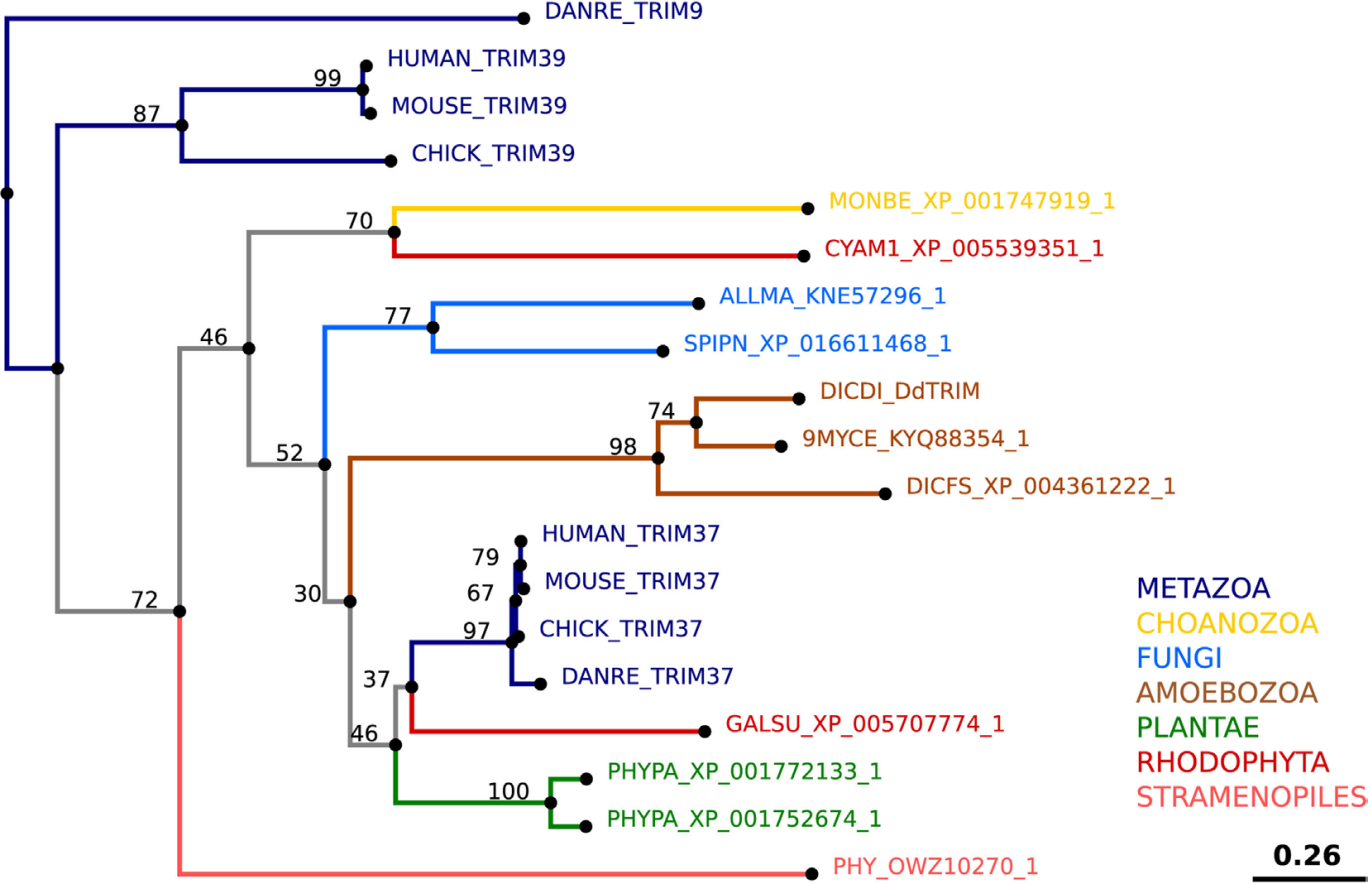
Theoretical phylogenetic relations of tripartite motif-containing protein (TRIM) proteins. The sequence of human TRIM37 was used to search for TRIM homologs among the non-redundant sequences in selected organisms using NCBI PSI-BLAST. A score of 2 × 10^−5^ was used as a threshold. Similarities of the selected sequences were determined using BLOSUM62 matrix and E-INS-i strategy (219). Sequences were manually curated using AliView software [(218); http://ormbunkar.se/aliview/], and the resulting final alignment was used to generate a neighbor joining phylogenetic tree (NJ, bootstrap 1,000×). TRIM39 was used as an outgroup.

Tripartite motif-containing protein homologs are found in multiple social amoeba species, and a single TRIM has been identified in *D. discoideum*, DdTRIM, which is an ortholog of human TRIM37 (Figure 10; Table 2) (http://dictybase.org). According to an accumulating amount of evidence, human TRIMs are emerging as critical regulators of cell-autonomous defenses. Particularly, human TRIM37 has been shown to restrict HIV-1 replication (322). At present, the role of DdTRIM remains unknown, and the presence of a single member of the TRIM superfamily early in evolution makes *D. discoideum* an interesting model to explore the primordial role of TRIM proteins before their expansion.

### Guanylate-Binding Proteins

The GBP proteins are IFN-gamma-inducible, immunity-related GTPases. Generally, the GBP proteins comprise a globular N-terminal domain and a C-terminal alpha-helical baculovirus inhibitor of apoptosis repeat (BIR) domain. Both the globular N-terminal domain, which confers GTPase activity, and the C-terminal BIR domain mediate protein–protein and protein–lipid interactions and contribute to nucleotide-dependent homotypic and heterotypic GBP protein assembly (323). In addition, BIR domains have been reported to act as caspase regulators and mediators of homotypic interactions (324). GTP binding to the GTPase activity domain allows dimer formation, and its hydrolysis enables conformational changes resulting in GBP tetramer formation. In vertebrates, the GBPs proteins have been linked to a multitude of innate immunity-related responses such as inflammasome activation and xenophagy (325). Essential for their function is their ability to oligomerize and to bind target endomembranes (323, 326, 327). In mouse macrophages, GBP2 recruitment to *Chlamydia trachomatis*- and *Toxoplasma gondii*-containing vacuoles correlates with their host-mediated lysis, underlying the importance of these large GTPases in the successful immune response against intracellular invaders (313, 328).

**Figure 11:**
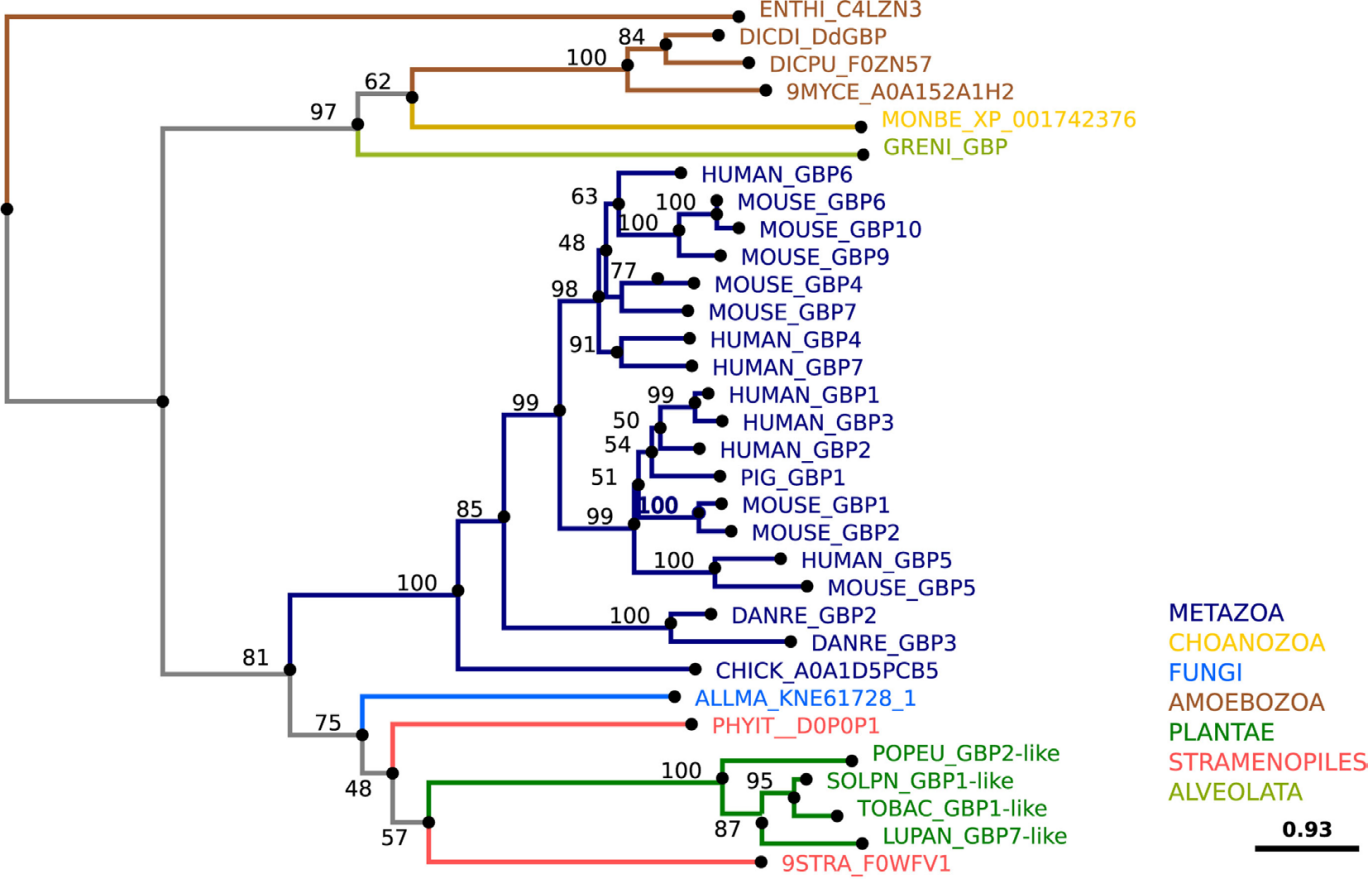
Theoretical phylogenetic relations of guanylate-binding proteins (GBPs). The sequences of human GBPs were used to search for GBP homologs among the non-redundant sequences in selected organisms using NCBI PSI-BLAST. A score of 2 × 10^−5^ was used as a threshold. Similarities of the selected sequences were determined using BLOSUM62 matrix and E-INS-i strategy (219). Sequences were manually curated using AliView software [(218); http://ormbunkar.se/aliview/], and the resulting final alignment was used to generate a neighbor joining phylogenetic tree (NJ, bootstrap 1,000×).

Multiple social amoeba species have homologs of GBPs, and a single GBP homolog has been identified in *D. discoideum*, DdGBP (Figure 11; Table 2). Its role remains to be elucidated. As a single GBP representative, it may allow a better understanding of the primordial and conserved role that GBPs play in MPS cells.

### Signal Transducers and Activators of Transcription

In humans, there are seven STATs, which have a unique N-terminus important for nuclear translocation and protein–protein interactions. This region is followed by a coiled-coil domain involved in nuclear export and regulation of tyrosine phosphorylation, a DNA-binding domain that mediates the recognition of sequences related to TTCN3–4GAA in the promoters of responsive genes, and an Src-homology (SH2) domain, which allows for specific recognition and docking to phosphotyrosines on cytokine receptors, Janus kinases (JAKs), and other STAT molecules. The C-terminus contains a divergent transactivator domain, which mediates STAT transcription factor transactivation *via* various cofactors (329).

Signal transducers and activators of transcription proteins are activated mainly by cytokines and growth factors. Binding of these signaling molecules to their receptors triggers receptor dimerization, allowing transphosphorylation and activation of receptor-associated JAKs. The JAKs also phosphorylate the cytoplasmic tails of the receptors, which promotes recruitment of the STAT proteins through their SH2 domain. The subsequent tyrosine phosphorylation of STATs results in the formation of homodimers and/or heterodimers and nuclear translocation, whereupon they bind to the promoter regions and initiate transcription of various immunity-related genes such as galectins, GBPs, NOXs, SQSTM1, TRIMs, and NDP52 (330). As part of their functional cycle, STATs shuttle between the cytosol and the nucleus (329). Mammalian STAT proteins act in a tissue-specific manner to regulate growth and development, various immunity-related processes, and cellular stress responses (331, 332). Gain- or loss-of-function mutations in components of the JAK–STAT signaling pathway have been associated with a broad range of innate-immune deficiencies and autoimmune diseases (333).

**Figure 12:**
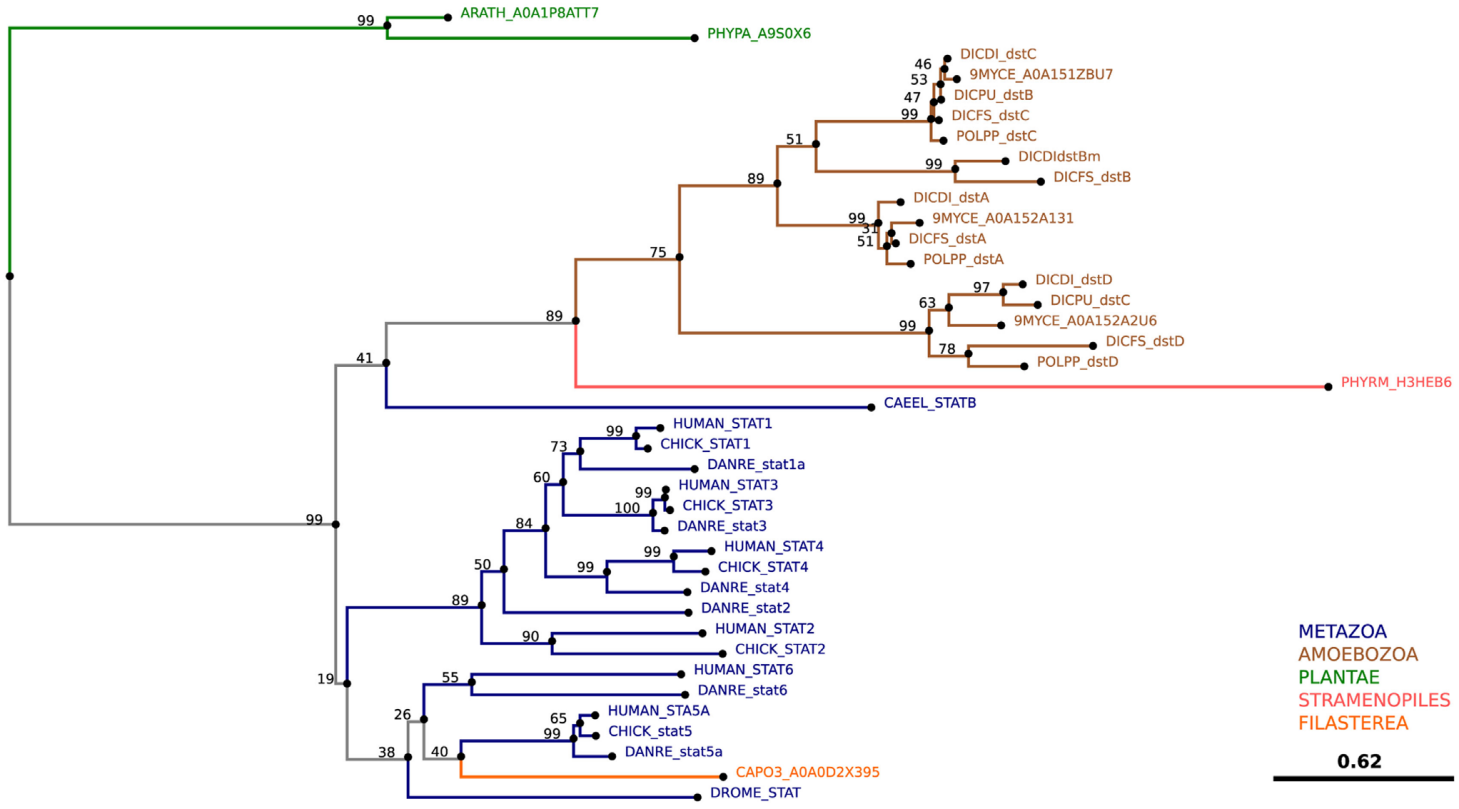
Theoretical phylogenetic relations of signal transducers and activators of transcription (STAT) family proteins. The sequence of the human STAT3 was used to search for STAT homologs among the non-redundant sequences in selected organisms using NCBI PSI-BLAST. A score of 2 × 10^−5^ was used as a threshold. Similarities of the selected sequences were determined using BLOSUM62 matrix and E-INS-i strategy (219). Sequences were manually curated using AliView software [(218); http://ormbunkar.se/aliview/], and the resulting final alignment was used to generate a neighbor joining phylogenetic tree (NJ, bootstrap 1,000×).

Multiple social amoeba species have STAT homologs, and four *D. discoideum* homologs of STATs have been identified, DstA, DstB, DstC, and DstD [reviewed in Ref. (334)] (Figure 12; Table 2). These proteins have a predicted SH2 domain (335), and their activity is regulated in part by tyrosine kinases (336, 337) and phosphatases (338, 339). Like mammalian STATs, *D. discoideum* STATs regulate growth and development in response to extracellular signaling molecules (335, 340–342) and are activated by cellular stresses (339, 343). Whether they also contribute to antimicrobial responses in *D. discoideum* is unknown, and addressing this question will provide insight into their possible roles in cell-autonomous defenses.

## Conclusion and Perspectives

*Dictyostelium discoideum* is a natural predator of bacteria and must contend with the fact that every meal is a potential infection. To survive this situation, it has evolved multiple mechanisms to generate a microbicidal environment within phagosomes and thus, phagocytosis, its means of nutrient acquisition, is simultaneously a major component of its defenses against infection. Autophagy, a pathway of nutrient reallocation, has also been incorporated into its defenses and is activated when microbes disrupt the phagosome and/or escape into the cytosol. Importantly, core components of these pathways are conserved in the specialized phagocytes of metazoans such as MPS cells. Consequently, *D. discoideum* is a relevant model to study the role of cell-autonomous defenses in the response of MPS cells to infection. Indeed, it has been used successfully to identify bacterial virulence factors and mechanisms by which bacteria interfere with phagosome maturation and autophagy.

Although one might question the need for model organisms in the CRISPR era of genomic editing, the use of *D. discoideum* has many advantages and is far from over. The generation of unbiased *D. discoideum* mutant libraries requires fewer financial and technical resources than creating a library of CRISPR-edited macrophages, and cultivation of this amoeba requires no special growth factors or cytokines. Moreover, growing *D. discoideum* cells respond to and phagocytose microbes without the need for prior activation. High-throughput mutant screens are thus more feasible and more accessible to laboratories with fewer financial resources. Similarly, the functions of individual bacterial or host factors in infection can be more readily and thoroughly assessed in *D. discoideum* due to its amenability to numerous research techniques. The knowledge gained can then be used to pose specific questions that can be answered by targeted studies in MPS cells. Primary macrophages or dendritic cells are often used to avoid the potential artifacts of immortalized cell lines. The use of *D. discoideum*, an organism compliant with the 3R initiative to reduce the use of animals (344), minimizes the need to sacrifice mice to procure these cells. Finally, given its lack of adaptive immunity, *D. discoideum* provides a context to study cell-autonomous defenses independent of other immune responses.

In addition to its importance as a model phagocyte, *D. discoideum* is also important from an ecological perspective. It is a key component of a complex network of bacteria, fungi, protists, and insects in the soil. The chemical language of this network is a potential source of natural products (345–348). Interactions between *D. discoideum* and bacteria can be symbiotic (349–351), and, as a reservoir and training ground for amoeba-resistant microbes, it can provide clues to understand and identify emerging pathogens. The structure of fruiting bodies appears to be evolutionarily optimized to be spread by insects (352), and insect-mediated transmission of fruiting bodies containing infectious *Bordetella bronchiseptica*, a respiratory pathogen of small mammals, has been demonstrated (353).

Many questions regarding interactions between intracellular pathogens and MPS cells remain unanswered, and *D. discoideum* still has much to teach us. For example, other than detecting disruption of the phagosome membrane, how *D. discoideum* “knows” it is infected is unknown. It might sense bacteria with PRRs, but, unless the bacteria are in the cytosol, this would not necessarily distinguish food bacteria that are readily killed from pathogenic bacteria. Given that its defense and nutrient pathways are intertwined, one possibility is that *D. discoideum* senses dysregulation of metabolism due to nutrient deprivation or imbalance. Another possibility is the recognition of DNA damage caused by bacterial toxins or excessive ROS production. Understanding how *D. discoideum* senses infection will open new avenues to explore in MPS cells. Other studies will continue to reveal the strategies used by microbes to resist being killed in the phagosome or by autophagy, to delineate the contributions of ROS to antimicrobial responses, and to elucidate the complexities of metal poisoning and nutritional immunity. Characterization of the *D. discoideum* versions of microbial restriction factors discovered in MPS cells will provide insight into their function and further establish the model. The future is bright for this fascinating and versatile amoeba.

## Author Contributions

JDD contributed ideas, wrote sections, created figures, edited, and compiled the manuscript. CBo, CBa, LR, LHL, EC-M, and ATL-J contributed ideas, wrote sections, and created figures. TS contributed ideas and edited.

## Conflict of Interest Statement

The authors declare that the research was conducted in the absence of any commercial or financial relationships that could be construed as a potential conflict of interest.
