# Supplementary material for: Eat Prey, Live: Dictyostelium discoideum As a Model for Cell-Autonomous Defenses
Source: Front Immunol. 2018 Jan 4;8:1906. doi: 10.3389/fimmu.2017.01906 (PMC5758549; doi:10.3389/fimmu.2017.01906)
Supplement: Supplementary file 1 [file Data_Sheet_1.zip › Supplementary Material.docx]

Supplementary Material

Eat prey, live: *Dictyostelium discoideum* as a model for cell-autonomous defenses

**Joe Dan Dunn*, Cristina Bosmani, Caroline Barisch, Lyudmil Raykov, Louise H. Lefrançois, Elena Cardenal-Muñoz, Ana Teresa López-Jiménez, and Thierry Soldati**

*** Correspondence:** Joe Dan Dunn: joedan.dunn@unige.ch

# Supplementary Data

The supplementary data include the sequence files used to create the alignments for the phylogenetic trees presented as figures in the article.

Figure 4. File: ZIP_tree_alignment.txt

Figure 5. File: ZnT_tree_alignment.txt

Figure 9. File: TRAF_alignment.txt

Figure 10. File: TRIM_alignment.txt

Figure 11. File: GBP_alignment.txt

Figure 12. File: STAT_alignment.txt

The alignments can be viewed using AliView (<http://www.ormbunkar.se/aliview/>)

Reference: Larsson, A. (2014). AliView: a fast and lightweight alignment viewer and editor for large data sets. *Bioinformatics*30(22): 3276-3278. <http://dx.doi.org/10.1093/bioinformatics/btu531>
